# A Molecular Electron Density Theory Study of the Reactivity of Azomethine Imine in [3+2] Cycloaddition Reactions

**DOI:** 10.3390/molecules22050750

**Published:** 2017-05-06

**Authors:** Luis R. Domingo, Mar Ríos-Gutiérrez

**Affiliations:** Department of Organic Chemistry, University of Valencia, Dr. Moliner 50, E-46100 Burjassot, Valencia, Spain; rios@utopia.uv.es

**Keywords:** azomethine imine, [3+2] cycloaddition reactions, molecular electron density theory, conceptual density functional theory, electron localisation function, bonding evolution theory, electron density, molecular mechanisms, chemical reactivity

## Abstract

The electronic structure and the participation of the simplest azomethine imine (AI) in [3+2] cycloaddition (32CA) reactions have been analysed within the Molecular Electron Density Theory (MEDT) using Density Functional Theory (DFT) calculations at the MPWB1K/6-311G(d) level. Topological analysis of the electron localisation function reveals that AI has a *pseudoradical* structure, while the conceptual DFT reactivity indices characterises this three-atom-component (TAC) as a moderate electrophile and a good nucleophile. The non-polar 32CA reaction of AI with ethylene takes place through a one-step mechanism with moderate activation energy, 8.7 kcal·mol^−1^. A bonding evolution theory study indicates that this reaction takes place through a non-concerted [2n + 2τ] mechanism in which the C–C bond formation is clearly anticipated prior to the C–N one. On the other hand, the polar 32CA reaction of AI with dicyanoethylene takes place through a *two-stage one-step* mechanism. Now, the activation energy is only 0.4 kcal·mol^−1^, in complete agreement with the high polar character of the more favourable regioisomeric transition state structure. The current MEDT study makes it possible to extend Domingo’s classification of 32CA reactions to a new *pseudo(mono)radical* type (*pmr-type*) of reactivity.

## 1. Introduction

**Scheme 1:**
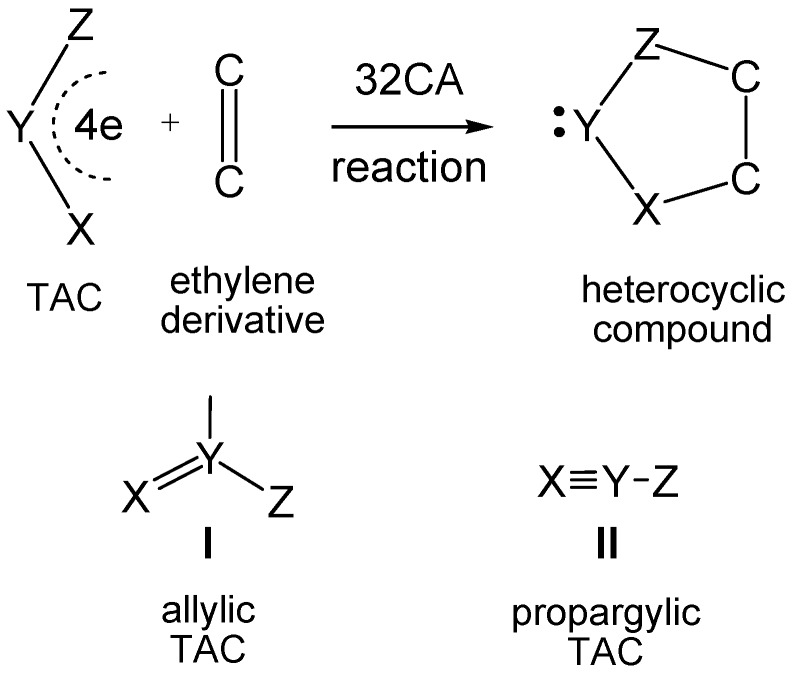
Construction of five-membered heterocyclic compounds by a 32CA reaction, and classification of TACs by structure.

[3+2] cycloaddition (32CA) reactions emerged as a powerful synthetic tool for the construction of five-membered heterocyclic compounds [1,2]. Although these reactions have been experimentally known since the end of the 19th century, it was Huisgen who, in 1961, defined them as “1,3-dipolar cycloadditions” [3,4]. These reactions are bimolecular in nature and involve the 1,3-addition of an ethylene derivative to a three-atom-component (TAC) (see Scheme 1). TACs can be structurally classified into two categories: allylic type (A-TAC) and propargylic type (P-TAC) structures [5,6]. While A-TACs such as **I** are bent, P-TACs such as **II** have a linear structure (see Scheme 1).

**Scheme 2:**
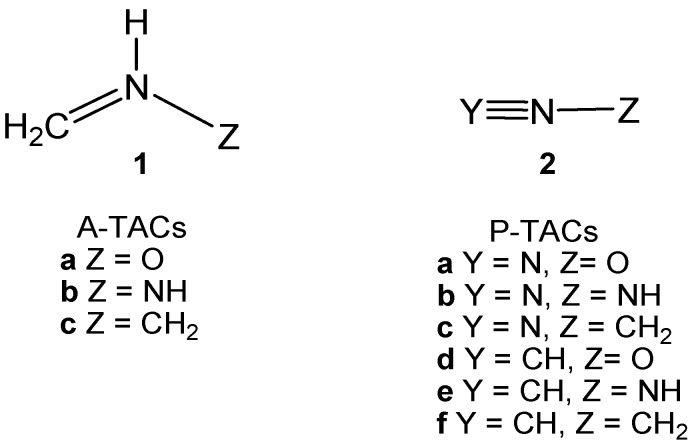
Nine TACs studied by Houk [7,8].

To explain the reactivity of TACs in 32CA reactions, Houk introduced, in 2007, a distortion/interaction energy model (DIEM) in which the activation barrier is divided into two additive terms: $\Delta E_{d}^{\neq }$, called *distortion* energy, and $\Delta E_{i}^{\neq }$, called *interaction* energy [7,8]. The applicability of this model was checked in 32CA reactions of nine different TACs, three A-TACs **1a**–**c** and six P-TACs **2a**–**f**, with ethylene **3** and acetylene **4** (see Scheme 2) [7,8].

**Figure 1:**
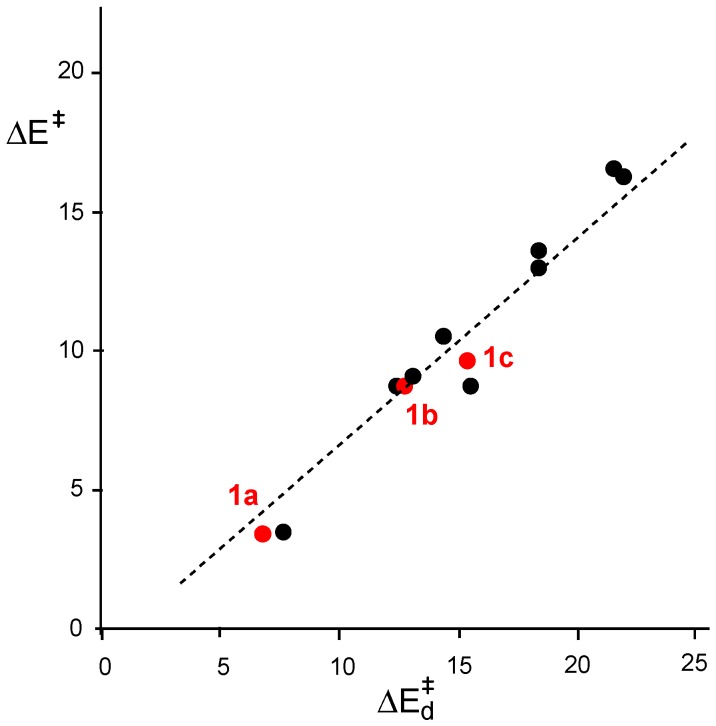
Plot of B3LYP/6-31G(d) activation energies ∆*E^≠^* vs. distortion energies: $\Delta E_{d}^{\neq }$, in kcal·mol^−1^, for Houk’s 32CA reactions of TACs **1** and **2** with ethylene **3** and acetylene **4** [7,8]. The position of TACs **1a**–**c** is marked in red (see Scheme 4).

Houk found that the computed B3LYP/6-31G(d) activation enthalpies correlated very nicely with the distortion energies: ∆*E^≠^* = 0.74 × $\Delta E_{d}^{\neq }$ − 0.78 kcal·mol^−1^ (R^2^ = 0.97) (see Figure 1). He concluded that the *distortion energy* of the reagents towards the transition state structure (TS) *is the major factor controlling the reactivity* differences of TACs. This finding, which can be considered a computational assertion of Hammond’s postulate established in 1955 [9], does not resolve the question why activation energies depend on geometries, which are the result of the distribution of the molecular electron density. In addition, the partition of the TS geometries into two separated fragments has no physical significance within Density Functional Theory [10] (DFT), since in this quantum chemical theory the energy is a functional of the electron density and the external potential, i.e., the nuclei positions. Consequently, the energy of the two separated fragments cannot be correlated with the energy of the TS because each of them loses the external potential created by the other fragment [11].

Very recently, Domingo has proposed a new reactivity theory in organic chemistry, namely, Molecular Electron Density Theory [12] (MEDT), in which changes in the electron density along an organic reaction, and not molecular orbital (MOs) interactions as proposed by the Frontier Molecular Orbital (FMO) theory [13], are responsible for its feasibility.

**Scheme 3:**
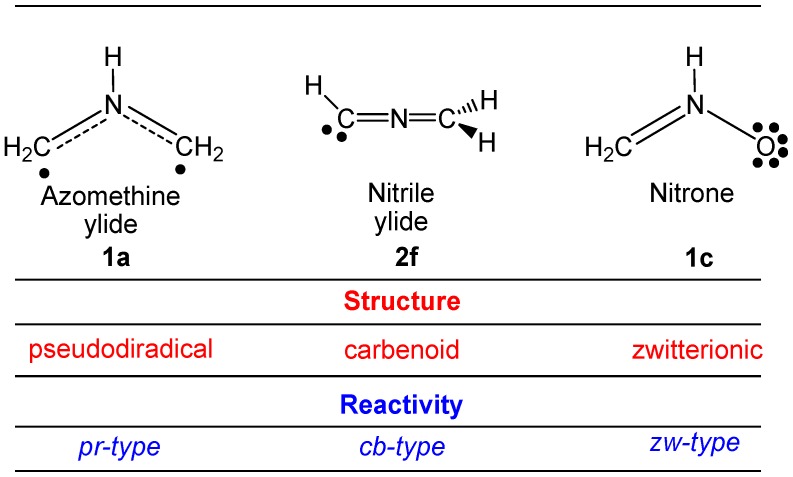
Electronic structure of TACs and the proposed reactivity types in 32CA reactions.

Several MEDT studies devoted to understanding the reactivity of TACs participating in 32CA reactions have allowed establishing a very good correlation between their electronic structures and reactivities. Accordingly, depending on the electronic structure of the TAC, i.e., *pseudodiradical* (typically an azomethine ylide (AY) **1a**) [14], carbenoid (typically a nitrile ylide **2f**) [15] or zwitterionic (typically a nitrone (Ni) **1c**), the 32CA reactions towards ethylene **3** have been classified into *pseudodiradical*-type (*pr-type*) [16], carbenoid-type (*cb-type*) [17] and zwitterionic-type (*zw-type*) [16] reactions (Scheme 3). The reactivity trend decreases in the order *pseudodiradical* > carbenoid > zwitterionic, in such a manner that while *pr-type* 32CA reactions take place easily through earlier TSs even with a very low polar character [16,18], *zw-type* 32CA reactions demand the adequate nucleophilic/electrophilic activations to take place [16,17,19]. Note that the feasibility of the three reactivity types depends on the polar character of the reaction, i.e., the nucleophilic/electrophilic interactions taking place at the TSs; the more polar the reaction, the faster the reaction.

**Scheme 4:**
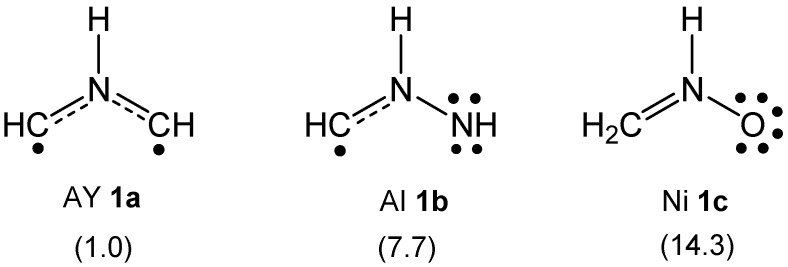
Series of CH_2_=NH–X (X = CH_2_, NH, O) A-TACs **1a**–**c**. In parentheses, MPWB1K/6-311G(d) activation energies, in kcal·mol^−1^ and with respect to the corresponding molecular complexes (MCs), associated with the 32CA reactions with ethylene **3**.

AY **1a**, azomethine imine (AI) **1b** and Ni **1c** constitute a series of three CH_2_=NH–X (X = CH_2_, NH, O) A-TACs in which the terminal X atom changes along the C, N, and O elements of the second arrow (see Scheme 4). In this short series of TACs, the activation energy associated with the 32CA reactions with ethylene **3** increases as the electronegativity of the atom X increases in the following order C < N < O (see Scheme 4) [16]. Interestingly, while the simplest AY **1a** has a *pseudodiradical* structure, [14] Ni **1c** has a zwitterionic one [20] (see Scheme 3). This behaviour causes these TACs to have a different reactivity towards ethylene **3** in 32CA reactions, i.e., the 32CA reaction involving AY **1a** is a *pr-type* reaction presenting a very low activation energy, 1.0 kcal·mol^−1^, while that involving Ni **1c** is a *zw-type* reaction with a high activation energy of 14.3 kcal·mol^−1^ (see Scheme 4). Note that it is expected that the reactivity of AI **1b**, which presents an activation energy towards ethylene **3** of 7.7 kcal·mol^−1^, will be different to that of AY **1a** and Ni **1c**.

**Scheme 5:**
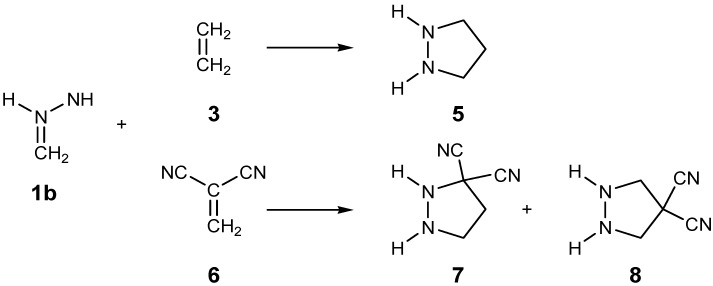
32CA reactions of AI **1b** with ethylene **3** and DCE **6**.

Considering that the simplest AI **1b** has a different activation energy towards ethylene **3** from that shown by AY **1a** and Ni **1c**, two TACs with a different electronic structure (see Scheme 3), an MEDT study of the 32CA reactions of the simplest AI **1b** with ethylene **3** and with electron-deficient (ED) dicyanoethylene (DCE) **6**, a strongly electrophilic ethylene, is herein carried out to establish the structure and reactivity of this TAC (see Scheme 5). Together with an electron localisation function (ELF) characterisation of the electronic structure of the simplest AI **1b**, a Bonding Evolution Theory [21] (BET) study of both reactions is performed in order to characterise the molecular mechanisms and to explain the activation energies implied in these cycloadditions.

## 2. Results and Discussion

The present theoretical study is divided into six parts: (i) an analysis of the electronic structure of AI **1b** is performed; (ii) the Conceptual DFT (CDFT) reactivity indices at the ground state (GS) of the reagents are analysed in order to predict the reactivity and regioselectivity in these 32CA reactions; (iii) the energy profiles associated with the 32CA reactions of AI **1b** with ethylene **3** and DCE **6** are studied; (iv) a BET study of the 32CA reaction of AI **1b** with ethylene **3** is performed to characterise the molecular mechanism of this cycloaddition; (v) an ELF topological analysis of the C–C and N–C bond formation processes along the polar 32CA reaction between AI **1b** and DCE **6** is carried out; and (vi) based on the electronic structure of AI **1b** and its reactivity towards ethylene **3**, a new type of reactivity in 32CA reactions is proposed.

### 2.1. ELF Topological Analysis and Natural Population Analysis (NPA) of AI ***1b***

**Figure 2:**
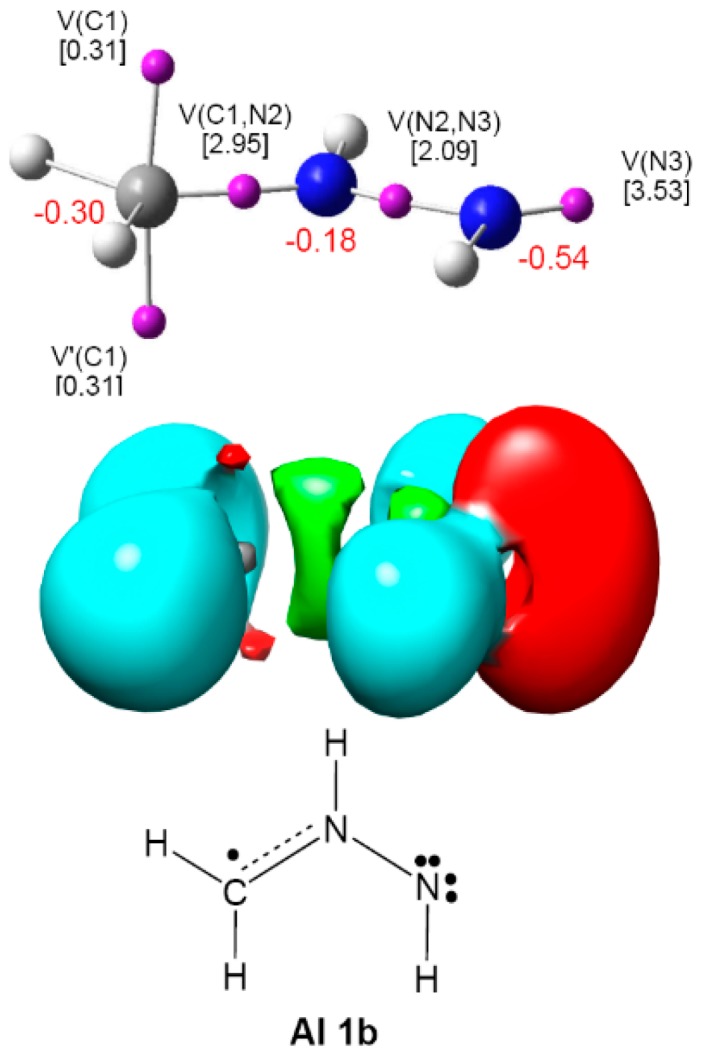
Representation of ELF attractors together with valence basin populations, natural atomic charges (in red) in average number of electrons (e), ELF localisation domains and the proposed Lewis structure for AI **1b**.

As the reactivity of the TACs has been correlated with their electronic structure [16,17], an ELF topological analysis of the simplest AI **1b** was first performed in order to characterise the electronic structure of this TAC and thus, to predict its reactivity in 32CA reactions. ELF attractors, including the valence basin populations, the natural atomic charges of C and N atoms, ELF localisation domains and the proposed Lewis structure arising from the ELF topological analysis for AI **1b**, are shown in Figure 2.

ELF topological analysis of the simplest AI **1b** shows the presence of two monosynaptic basins, V(C1) and V’(C1), integrating a total electron density of 0.62 e, two disynaptic basins, V(C1,N2) and V(N2,N3), with electron populations of 2.95 e and 2.09 e, and one V(N3) monosynaptic basin integrating 3.53 e. These ELF basins are related with the presence of a C1 *pseudoradical* centre, a C1–N2 bonding region integrating ca. 3 e, an N2–N3 single bond and an N3 non-bonding electron density (see the ELF-based Lewis structure of AI **1b** in Figure 2).

According to the Lewis structures, V(C) monosynaptic basins integrating ca. 1 e are associated to *pseudoradical* centres [14,18], while those integrating ca. 2 e in neutral molecules are associated to carbenoid centres [17]. TACs presenting two *pseudoradical* centres have been classified as *pseudodiradical* TACs [16], while those presenting a carbenoid centre have been classified as carbenoid TACs [17]. Finally, TACs that neither present *pseudoradical* nor carbenoid centres have been classified as zwitterionic TACs [16]. Consequently, ELF topological analysis of the electronic structure of the simplest AI **1b** indicates that this TAC, which presents a *pseudoradical* structure, does not have the electronic structure of any of the three representative *pseudodiradical*, carbenoid and zwitterionic TACs given in Scheme 3.

After the establishment of the bonding pattern of AI **1b** based on the ELF topological analysis, the charge distribution was analysed. The natural atomic charges, obtained through an NPA, are shown in Figure 2. As can be observed, the three atoms belonging to this TAC present negative charges: −0.30 e (C1), −0.18 e (N2) and −0.54 e (N3), while the hydrogen atoms support the positive charges. This charge distribution is in complete disagreement with the commonly accepted 1,2-zwitterionic structure given for AIs in which a positive charge and a negative charge are entirely located at the N2 and N3 nitrogen atoms [3,22].

Thus, while NPA reveals that this TAC does not have a 1,2-zwitterionic Lewis structure, ELF topological analysis of the electron density of AI **1b** permits establishing a *pseudoradical* electronic structure with a *pseudoradical* centre at the C1 carbon atom. The distinct electronic structure of AI **1b** with respect to that of the *pseudodiradical*, carbenoid and zwitterionic structures given in Scheme 3 justifies the different reactivity of these TACs (see Scheme 4), and therefore, the establishment of a new reactivity model in 32CA reactions.

### 2.2. Analysis of the CDFT Reactivity Indices at the GS of the Reagents

Global reactivity indices defined within CDFT [23,24] are powerful tools to explain the reactivity in cycloaddition reactions. Since the global electrophilicity and nucleophilicity scales are given at the B3LYP/6-31G(d) level, the present analysis has been performed at this computational level. The global indices, namely, the electronic chemical potential (μ), the chemical hardness (η), the electrophilicity (ω) and the nucleophilicity (*N*), for AI **1b**, ethylene **3** and DCE **6**, as well as the *pr* index of A-TACs **1a**–**c**, are presented in Table 1.

As shown in Table 1, the electronic chemical potential μ of AI **1b**, −2.70 eV, is higher than that of ethylene **3**, −3.37 eV, and DCE **6**, −5.64 eV. Consequently, along polar 32CA reactions, the global electron density transfer [25] (GEDT) will take place from AI **1b** toward ethylene **3** or DCE **6**; however, note that ethylene **3** has no tendency to participate in polar processes.

Along a polar reaction, there is an electron density transfer from the nucleophilic to the electrophilic species, which is measured by the GEDT [25] value computed at the TS of the reaction; the larger the GEDT at the TS, the more polar the reaction. Note that the GEDT concept comes from the observation that the electron density transfer taking place from the nucleophile to the electrophile along a polar reaction is not a local process, but a global one involving the two interacting frameworks and depending on the electrophilic/nucleophilic interactions taking place between them [25]. It should be emphasised here that this global property is lost with the molecular fragmentation carried out in Houk’s DIEM [11].

The simplest AI **1b** has an electrophilicity ω index of 0.72 eV and a nucleophilicity *N* index of 3.92 eV, being classified as a marginal electrophile [26] and as a strong nucleophile [27]. The high electron density accumulated in the three heavy atoms belonging to AI **1b** could account for the high nucleophilic character of this TAC (see NPA in Section 2.1). Consequently, AI **1b** will participate only as a strong nucleophile in polar 32CA reactions.

Analysis of the reactivity indices of the three A-TACs given in Scheme 4 shows that the electrophilicity ω index increases and the nucleophilicity *N* index decreases as the electronegativity of the terminal X atom increases in the following order C < N < O; in any case, the three A-TACs are strong nucleophiles.

In order to characterise the participation of TACs in a *pr-type* 32CA reaction, the *pseudodiradical* (*pr*) index, has recently been introduced [16]. A-TACs with *pr* values larger than 0.90 can be related to species having a very soft character, i.e., with low hardness η values, and low stabilised frontier electrons, i.e., low ionisation potential, participating in *pr-type* 32CA reactions, while A-TACs with low *pr* values should participate in *zw-type* 32CA reactions. A-TACs AY **1a**, AI **1b** and Ni **1c** have *pr* values of 1.13, 0.78 and 0.53 (see Table 1), indicating that AI **1b** will not present a *pr-type* reactivity in 32CA reactions.

Polar cycloaddition reactions require the participation of good electrophiles and good nucleophiles. Ethylene **3** is one of the poorest electrophilic, ω = 0.73 eV, and nucleophilic, *N* = 1.86 eV, species involved in cycloaddition reactions, being classified as a marginal electrophile and as a marginal nucleophile. Consequently, ethylene **3** cannot participate in polar reactions. Substitution of two gem hydrogen atoms in ethylene **3** by two electron-withdrawing –CN groups in DCE **6** considerably increases the electrophilicity ω index to 2.82 eV and decreases the nucleophilicity *N* index to 0.65 eV. Consequently, DCE **6** will participate only as a strong electrophile in polar 32CA reactions. Given the high nucleophilic character of AI **1b** and the strong electrophilic character of DCE **6**, it is expected that the 32CA reaction between AI **1b** and DCE **6** will have a high polar character.

**Figure 3:**
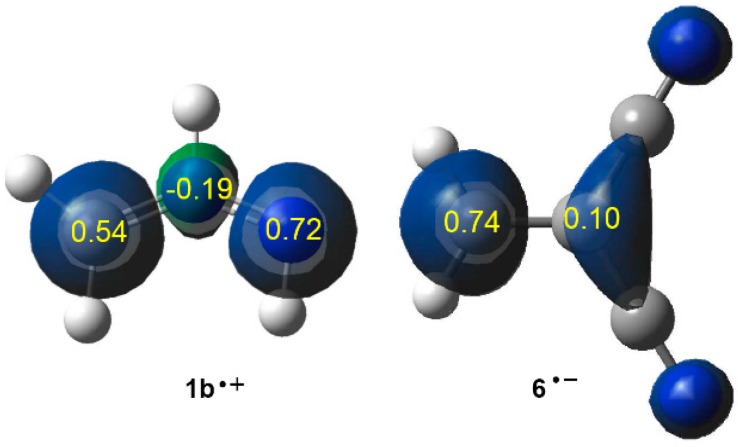
Three-dimensional (3D) representations of the atomic spin densities (ASD) of the radical cation **1b^·+^** and the radical anion **6^·−^**, together with the nucleophilic *P_k_^-^* Parr functions of AI **1b** and the electrophilic *P_k_^+^* Parr functions of DCE **6**.

By approaching a non-symmetric electrophilic/nucleophilic pair along a polar or ionic process, the most favourable reactive channel is that associated with the initial two-centre interaction between the most electrophilic centre of the electrophile and the most nucleophilic centre of the nucleophile. Recently, Domingo proposed the nucleophilic *P_k_^-^* and electrophilic *P_k_^+^* Parr functions [28], derived from the changes of spin electron-density reached via the GEDT process from the nucleophile to the electrophile, as a powerful tool in the study of the local reactivity in polar or ionic processes. Accordingly, the nucleophilic *P_k_^-^* Parr functions of AI **1b** and the electrophilic *P_k_^+^* Parr functions of DCE **6** were analysed in order to characterise the most nucleophilic and electrophilic centres of the species involved in this polar 32CA reaction (see Figure 3).

Analysis of the nucleophilic *P_k_^-^* Parr functions at the reactive sites of AI **1b** indicates that both the C1 carbon atom, with a *P_k_^-^* value of 0.54, and the N3 nitrogen atom, with a *P_k_^-^* value of 0.72, are nucleophilically activated, the latter more than the former. Note that the N2 nitrogen atom is nucleophilically deactivated, possessing a negative *P_k_^-^* value of −0.19. On the other hand, the electrophilic *P_k_^+^* Parr functions at the reactive sites of DCE **6** indicate that the more electrophilic entre is the C4 carbon atom, possessing the maximum value of *P_k_^+^* = 0.74.

Therefore, it can be predicted that along a polar reaction the most favourable electrophile-nucleophile interaction along the nucleophilic attack of AI **1b** on DCE **6** will take place between the most nucleophilic centre of AI **1b**, the N3 nitrogen atom, and the most electrophilic centre of DCE **6**, the C4 carbon atom.

### 2.3. Study of the Reaction Channels Associated with the 32CA Reactions of AI ***1b*** with Ethylene ***3*** and DCE ***6***

#### 2.3.1. 32CA Reaction Involving Ethylene **3**

**Scheme 6:**
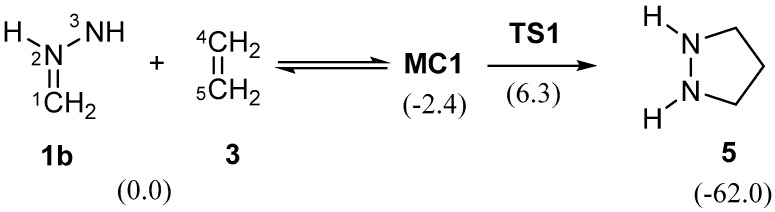
32CA reaction between AI **1b** and ethylene **3**. MPWB1K/6-311G(d) relative energies, in kcal·mol^−1^, are given in parentheses.

**Figure 4:**
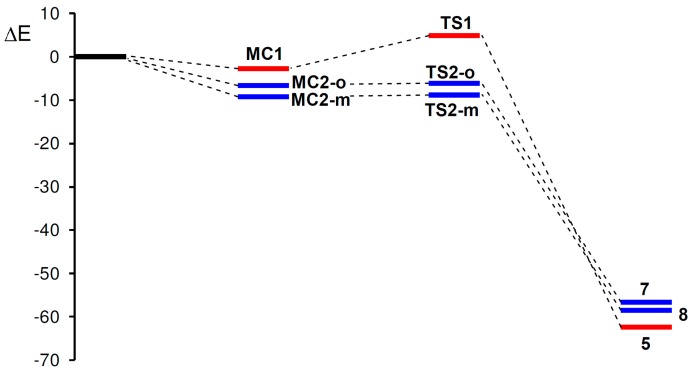
Energy profile (∆*E*, in kcal·mol^−1^) of the 32CA reaction of AI **1b** with ethylene **3**, in red, and with DCE **6**, in blue.

Due to the symmetry of ethylene **3**, the reagents, **1b** and **3**, one MC, **MC1**, only one TS, **TS1**, and the corresponding pyrazolidinone **5** were located and characterised; consequently, the 32CA reaction takes place through a one-step mechanism (see Scheme 6). The MPWB1K/6-311G(d) total and relative energies of the stationary points involved in the 32CA reaction of AI **1b** with ethylene **3** are given in Table S1 of the Supplementary Materials and Scheme 6, respectively, while the energy profile is graphically represented in red in Figure 4.

The reaction between AI **1b** and ethylene **3** begins with the formation of **MC1**, which is slightly stabilised by only 2.4 kcal·mol^−1^ with respect to the separated reagents (see Figure 4). From **MC1**, the activation energy associated with **TS1** is 8.7 kcal·mol^−1^, the reaction being exothermic by 59.6 kcal·mol^−1^. This activation energy is found between that associated with the non-polar 32CA reaction of AY **1a** with ethylene **3**, 1.0 kcal·mol^−1^, a *pr-type* reaction, and that associated with the non-polar 32CA reaction of Ni **1c** with ethylene **3**, a *zw-type* reaction, 14.3 kcal·mol^−1^ (see Scheme 4).

**Figure 5:**
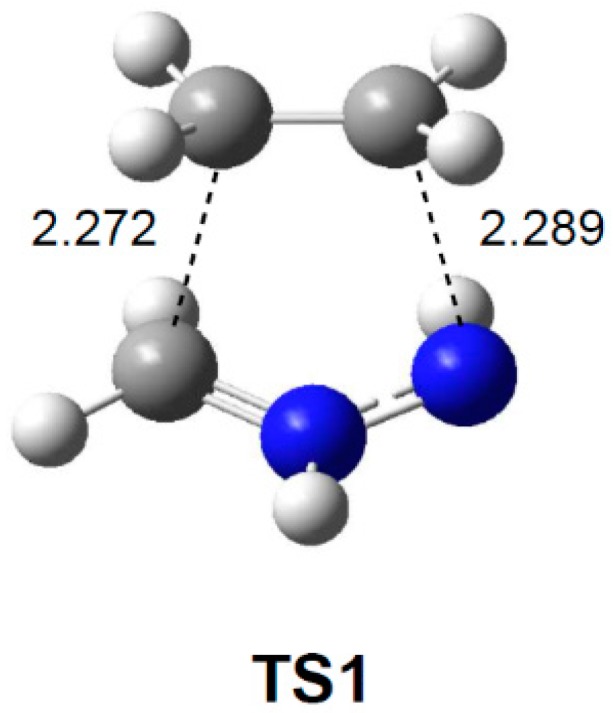
MPWB1K/6-311G(d) geometry of the TS associated with the 32CA reaction between AI **1b** and ethylene **3**. Distances are given in Angstroms, Å.

The geometry of **TS1** is displayed in Figure 5. At **TS1**, the distances between the C1 and C5, and the N3 and C4 interacting atoms are 2.272 and 2.289 Å, respectively. It has been well established that the formation of C–C single bonds takes place in the short distance range of ca. 1.9–2.0 Å [25], while several studies have shown that formation of C–N single bonds begins at shorter distances, ca. 1.7 Å [29]. Therefore, despite the geometrical symmetry of **TS1**, these parameters suggest an asynchronous bond formation process in which the C1–C5 bond formation is more advanced than the N3–C4 one.

The electronic nature of the 32CA reaction between AI **1b** and ethylene **3** was analysed by computing the GEDT [25] at the corresponding TS. Cycloadditions with GEDT values near 0.0 e correspond to non-polar processes, whereas values higher than 0.2 e correspond to polar processes. In gas phase, the GEDT that fluxes from the AI moiety towards the ethylene one is 0.10 e at **TS1**. This value indicates that this 32CA reaction has a low polar character. Interestingly, the slight GEDT computed at **TS1**, whose direction is in agreement with the analysis of the corresponding electronic chemical potential μ indices, can be rationalised as a delocalisation of the energetically destabilised electron density of the AI framework into the ethylene one, rather than a GEDT associated to a polar process [14]. Note that ethylene **2** has neither electrophilic nor nucleophilic character.

#### 2.3.2. 32CA Reaction Involving DCE **6**

**Scheme 7:**
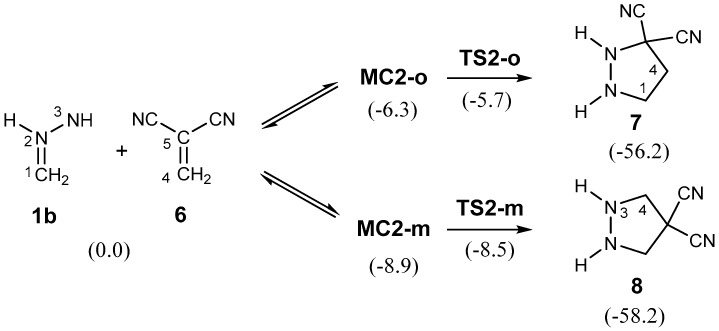
32CA reaction between AI **1b** and DCE **6**.

Due to the non-symmetry of AI **1b** and DCE **6**, this 32CA reaction can take place through two regioisomeric channels, the *ortho* and the *meta* (see Scheme 7), i.e., those associated with the initial formation of the C1–C4 and N3–C4 single bonds, respectively. A search for the stationary points involved in the two regioisomeric pathways allowed finding two MCs, **MC2-o** and **MC2-m**, two regioisomeric TSs, **TS2-o** and **TS2-m**, and the corresponding pyrazolidines **7** and **8**, which were properly characterised; consequently, the 32CA reaction takes place through a one-step mechanism (see Scheme 7). The MPWB1K/6-311G(d) total and relative energies of the stationary points involved in the 32CA reaction of AI **1b** with DCE **6** are given in Table S2 of the Supplementary Materials and Scheme 7, respectively, while the energy profile is graphically represented in blue in Figure 4.

When AI **1b** and DCE **6** gradually approach each other, the energy is reduced until the formation of two MCs located 6.3 (**MC2-o**) and 8.9 (**MC2-m**) kcal·mol^−1^ below the separated reagents takes place (see Figure 4). Further approach of both the AI and the DCE frameworks leads to the formation of the TSs, which are found 3.2 (**TS2-o**) and 0.4 (**TS2-m**) kcal·mol^−1^ above the more stable **MC2-m**. Note that both MCs are in thermodynamic equilibrium. Moreover, formation of pyrazolidines **7** and **8** from **MC2-m** becomes strongly exothermic by 47.3 and 49.3 kcal·mol^−1^, respectively. Some appealing conclusions can be drawn from these energy results: (i) the activation barrier associated to the more favourable **TS2-m** is 8.3 kcal·mol^−1^ lower than that associated to **TS1**, 8.7 kcal·mol^−1^; (ii) this 32CA reaction is highly regioselective, **TS2-o** being 2.8 kcal·mol^−1^ above **TS2-m**; and (iii) the strong exothermic character of this reaction makes the formation of pyrazolidines **7** and **8** irreversible.

**Figure 6:**
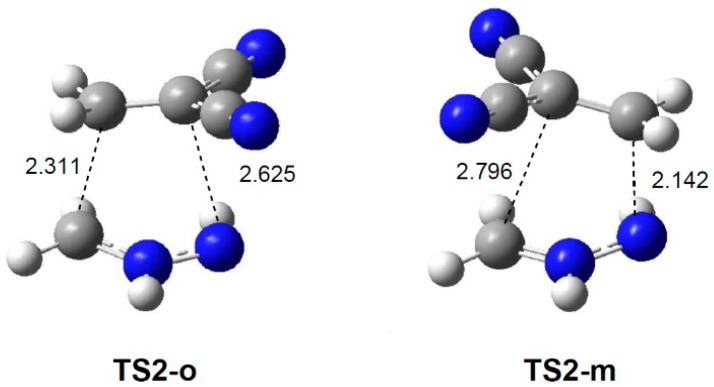
MPWB1K/6-311G(d) geometries of the TSs associated with the 32CA reaction between AI **1b** and DCE **6**. Distances are given in Angstroms, Å.

The geometries of the TSs associated with the 32CA reaction between AI **1b** and DCE **6** are displayed in Figure 6. At the *ortho* **TS2-o**, the distances between the C1 and C4, and the N3 and C5 interacting atoms are 2.311 and 2.625 Å, while at the *meta* **TS2-m**, the distances between the N3 and C4, and the C1 and C5 interacting atoms are 2.142 and 2.796 Å. Some appealing conclusions can be drawn from these geometrical parameters: (i) both TSs correspond to highly asynchronous single bond formation processes in which the formation at the β conjugated position of DCE **6** is more advanced than that at the α one; (ii) geometrically, the more favourable **TS2-m** is more advanced and more asynchronous than **TS2-o**; and (iii) the more favourable **TS2-m** is associated to the two-centre interaction between the most nucleophilic centre of AI **1b** and the most electrophilic centre of DCE **6**, in complete agreement with the analysis of the Parr functions (see Section 2.2).

In gas phase, the GEDT that fluxes from the AI framework towards the ethylene one is 0.25 e at **TS2-o** and 0.27 e at **TS2-m**. The GEDT at the more favourable **TS2-m** is slightly higher than that at **TS2-o**. These high values indicate that this 32CA reaction has a high polar character, in clear agreement with the very high nucleophilic character of AI **1b** and the high electrophilic character of DCE **6**, and account for the large decrease of the activation energy with respect to the non-polar 32CA reaction involving ethylene **3** via **TS1**.

### 2.4. BET Study of the 32CA Reaction of AI ***1b*** with ethylene ***3***

When trying to achieve a better understanding of bonding changes in organic reactions, the so-called BET [21] has proven to be a very useful methodological tool. This quantum-chemical methodology makes it possible to understand the bonding changes along a reaction path and, thus, to establish the nature of the electronic rearrangement associated with a given molecular mechanism [30].

**Figure 7:**
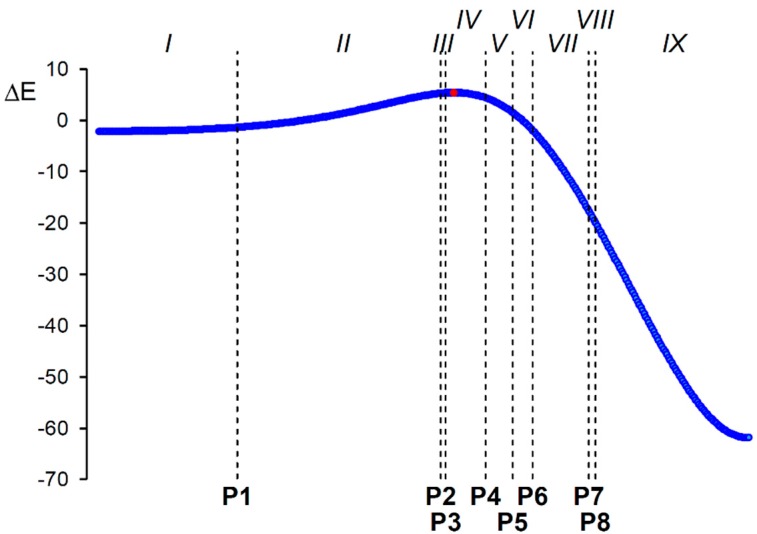
The nine phases, separated by black dashed lines, in which the IRC (in amu^1/2^·Bohr) associated with the non-polar 32CA reaction between AI **1b** and ethylene **3** is topologically divided. Relative energy variations, Δ*E*, with respect to the separated reagents, are given in kcal·mol^−1^. The red point indicates the position of **TS1**.

**Figure 8:**
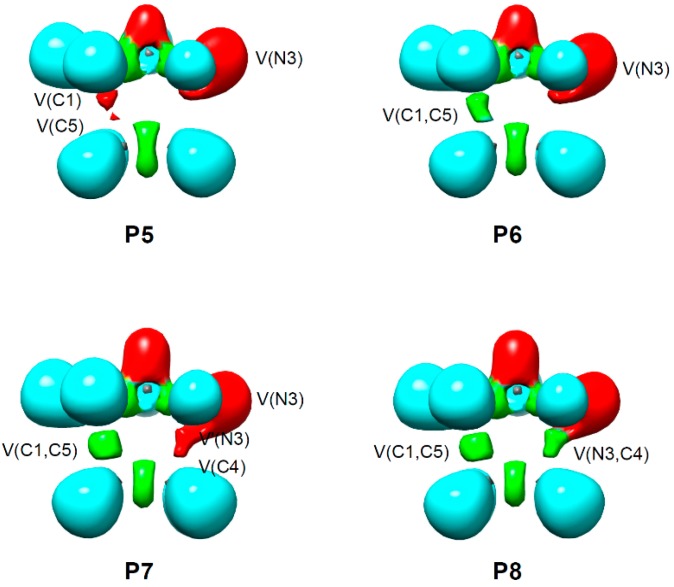
ELF localisation domains, represented at an isovalue of 0.74, for the points of the IRC defining *Phases VI–IX* involved in the formation of the C1–C5 and N3–C4 single bonds along the non-32CA reaction between AI **1b** and ethylene **3**.

In order to characterise the molecular mechanism of the non-polar 32CA reaction of AI **1b** with ethylene **3**, a BET study along the cycloaddition reaction was carried out. The complete BET study is provided in the Supplementary Materials. Some appealing conclusions can be drawn from this BET study: (i) the intrinsic reaction coordinate (IRC) associated with the 32CA reaction of the simplest AI **1b** with ethylene **3** is divided in nine differentiated phases, a behaviour that clearly indicates that the bonding changes along this one-step mechanism are non-concerted (see Figure 7); (ii) ELF topological analysis of **TS1** indicates that there is no bonding region between the N1 and C4, and the C3 and C4 interacting atoms, respectively; (iii) the moderate activation energy associated with this reaction, 8.7 kcal·mol^−1^, can be mainly associated with the rehybridisation of the C1 carbon from sp^2^ to sp^3^; (iii) formation of the first C3–C4 single bond takes place at a C–C distance of 2.03 Å through the C-to-C coupling of two C3 and C4 *pseudoradical* centres [25] (see points **P5** and **P6** in Figure 8); (iv) interestingly, while the C4 *pseudoradical* centre is generated along the reaction path through the depopulation of the C4–C5 double bond of ethylene **3**, the C3 *pseudoradical* centre is already present at the simplest AI **1b**; (v) formation of the second N1–C4 single bond takes place at an N–C distance of 1.92 Å through the C-to-N coupling of two N1 and C5 *pseudoradical* centres (see points **P7** and **P8** in Figure 7); (vi) formation of this C–N single bond is thus different to that found in the ketene-imine Staudinger reaction in which the first C–N single bond is formed through the donation of the electron density of the imine nitrogen lone pair to the ketene carbonyl carbon [29]; and (vii) the present BET study allows establishing the molecular mechanism of the non-polar 32CA reaction between the simplest AI **1b** and ethylene **3** and characterising it as a [2n + 2τ] process. The non-bonding 2n electrons involved in this 32CA reaction can be associated with the *pseudoradical* centre present at the C1 carbon and part of the electron density of the N3 nitrogen lone pairs of the simplest AI **1b**, while the 2τ electrons come from the C4–C5 double bond of ethylene **3**. Note that, in 1931, first Pauling [31] and later Slater [32] proposed that the C–C bonding region of ethylene **3** can be represented by two equivalent bonds named τ bonds. This electronic representation of C–C double bonds is in complete agreement with the ELF topological analysis of ethylene **3** in which the corresponding C–C bonding region is characterised by the presence of two equivalent V(C,C) disynaptic basins integrating ca. 2 e each one (see the Supplementary Materials).

### 2.5. ELF Topological Analysis of the C–C and N–C Bond Formation Processes along the Polar 32CA Reaction between AI ***1b*** and DCE ***6***. Understanding the Role of the GEDT

Recently, an ELF topological analysis of some relevant points involved in the formation of the new single bonds along the IRC associated with the experimental 32CA reaction of an AI derivative with *N*-vinyl tetrahydroindole allowed establishing that this reaction takes place through a *two-stage one-step* mechanism [33]. Herein, in order to investigate the bond formation processes along the polar 32CA reaction of AI **1b** with DCE **6** and to understand the low activation energies associated with the two *meta/ortho* regioisomeric channels, an ELF topological analysis of the corresponding stationary points and some relevant points along the IRC involved in the formation of the new C–C and N–C single bonds was performed. Note that these points were selected by a similar procedure to that used in the previous BET study (see Computational Methods). The complete ELF topological analysis is provided in the Supplementary Materials, while a summary of the most appealing conclusions is reported herein.

**Figure 9:**
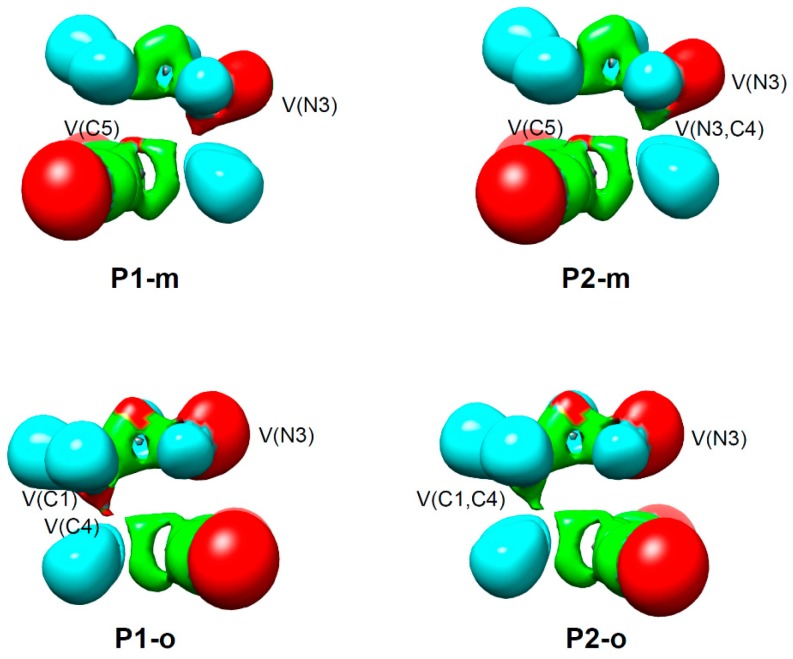
ELF localisation domains, represented at an isovalue of 0.68, for the selected points of the IRC involved in the formation of the first N3–C5 (*meta*) and C1–C4 (*ortho*) single bonds along the two *meta*/*ortho* reactive channel associated with the polar 32CA reaction between AI **1b** and DCE **6**.

Some appealing conclusions can be drawn from this ELF topological analysis along both *meta/ortho* regioisomeric channels: (i) in both reaction channels, the formation of the first single bond involves the most electrophilic centre of DCE **6**, the C4 carbon (see Figure 9); (ii) formation of the C–C single bonds along the two channels begins at C–C distances of 2.14 Å (*meta*) and 2.05 Å (*ortho*) through the C-to-C coupling of two C1 and C4/C5 *pseudoradical* centres (see **P1-o** and **P2-o** in Figure 9) [29]; (iii) interestingly, while along the more favourable *meta* channel the two C1 and C5 *pseudoradical* centres are created as the reaction progresses, the C1 *pseudoradical* centre is already present at *ortho* **MC2-o**; (iv) while along the more favourable *meta* channel the C5 *pseudoradical* centre created at the DCE framework participates more than the C1 one created at the AI moiety in the C–C bond formation process, along the *ortho* channel the C1 *pseudoradical* centre already present at **MC2-o** contributes more to the C–C bond formation; (v) conversely, the N–C bond formation takes place differently along both channels. Formation of the N–C single bond begins at N–C distances of 1.81 Å (*meta*) and 1.84 Å (*ortho*) through the donation of part of the non-bonding electron density of the N3 nitrogen to the C4 carbon along the *meta* channel (see **P1-m** and **P2-m** in Figure 9) or through the C-to-N coupling of two C5 and N3 *pseudoradical* centres along the *ortho* channel; (vi) both reaction channels present highly asynchronous bond formation processes, in agreement with the previous geometry analysis. The polar 32CA reaction between AI **1b** and DCE **6** proceeds through a *two-stage one-step* mechanism [33] in which the formation of the second bond begins when the first one is already formed by up to 95%; (vii) the bonding patterns of **TS2-o** and **TS2-m** are very similar to those of the corresponding MCs and, accordingly, the very low energy barriers relative to the corresponding MCs, 0.6 (**TS2-o**) and 0.4 (**TS2-m**) kcal·mol^−1^, can mainly be associated with slight electron density variations within the molecular system; (viii) therefore, these very low activation energies can be the consequence of the GEDT taking place at the polar TSs, which favours the polar 32CA reaction through an electronic stabilisation of both the nucleophile and electrophile frameworks; (ix) the energy difference between **TS2-o** and **TS2-m**, 2.8 kcal·mol^−1^, is likely to be associated to the higher stability of the electron density distribution at **TS2-m** than that at the *pseudoradical* structure of **TS2-o**.

The GEDT taking place at the TSs of the polar 32CA reaction between AI **1b** and DCE **6** does not only decrease the activation energy associated with the non-polar 32CA reaction involving ethylene **3**, but also modifies the molecular mechanism of the polar reaction making the most favourable reaction channel that involving the first N–C bond formation instead of the C–C one, in agreement with the analysis of the Parr functions. Note that, despite the *pseudoradical* character of the C1 carbon, the N3 nitrogen is the most nucleophilic centre of AI **1b** (see Section 2.2).

A comparative analysis between the BET study of the non-polar 32CA reaction involving ethylene **3** and the ELF topological analysis of the bond formation processes along the polar 32CA reaction involving DCE **6** makes it possible to understand the role of the GEDT in the polar process. Some appealing conclusions emerge from the corresponding comparative analysis: (i) while along the non-polar 32CA reaction of AI **1b** with ethylene **3** and the less favourable *ortho* channel of the polar 32CA reaction between AI **1b** and DCE **6** the most favourable interaction is that involving the C1 *pseudoradical* centre, along the more favourable *meta* channel of the polar 32CA reaction between AI **1b** and DCE **6** is that involving the interaction between the most nucleophilic and electrophilic centres of the reagents; (ii) thus, while the non-polar cycloaddition and the *ortho* channel of the polar reaction begin with the initial formation of the C–C single bond, the *meta* channel of the polar reaction begins with the initial formation of the N–C single bond. Consequently, both mechanisms are different; (iii) formation of the new single bonds is slightly asynchronous in the non-polar reaction but highly asynchronous in the polar reaction; (iv) unlike polar Diels–Alder reactions and polar *zw-type* 32CA reactions in which the GEDT favours the bonding changes at the reagents, i.e., the rupture of the double bonds [34], in the polar 32CA reaction between AI **1b** and DCE **6**, the GEDT provokes an electronic stabilisation of both the nucleophilic and the electrophilic frameworks at the TSs, decreasing the activation energies from 8.7 kcal·mol^−1^ (**TS1**) to 0.4 (**TS2-o**) and 3.2 (**TS2-m**) kcal·mol^−1^.

### 2.6. Understanding the Reactivity of AI ***1b*** Possessing a Carbon Pseudoradical Centre

ELF topological analysis of several TACs have shown that unlike butadiene **9**, which presents a conjugated C–C double bond Lewis structure, TACs have very complex electronic structures that cannot be usually represented by a simple Lewis structure. On the other hand, unlike the non-polar Diels–Alder reaction between butadiene **9** and ethylene **3**, which has a high activation energy of ca. 25 kcal·mol^−1^ [35], the non-polar 32CA reactions between TACs and ethylene **3** have activation energies that range from 1 to 15 kcal·mol^−1^ [7,8]. Note that ethylene **3** is a poor electrophile and a poor nucleophile that cannot participate in polar reactions.

**Figure 10:**
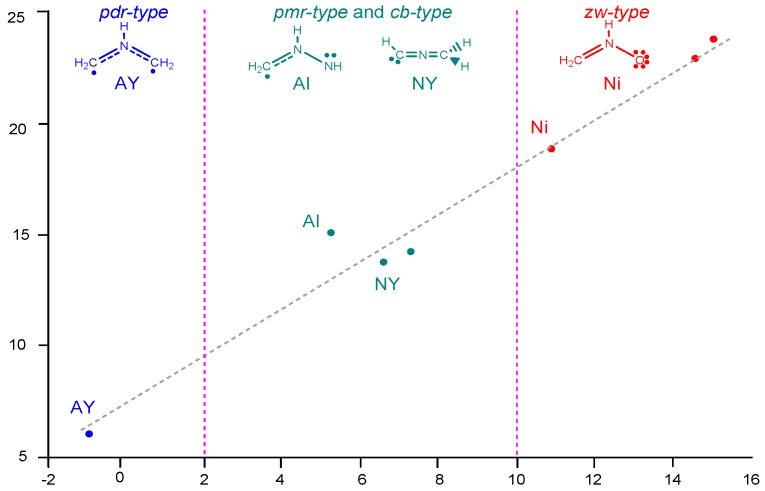
Plot of the MPWB1K/6-311G(d) relative energies ∆*E*^≠^ vs. the distortion energies ∆$E_{d}^{\neq }$, in kcal·mol^−1^, for Houk’s 32CA reactions of TACs **1** and **2**, having at least one carbon atom, with ethylene **3** [7,8].

As has been aforementioned in the Introduction, a good correlation between the electronic structures of TACs and the activation energies involved in the non-polar 32CA reactions towards ethylene **3**, i.e., their reactivity, has been found [16,17]. Thus, while zwitterionic TACs such as Ni **1c**, which must break the C–N double bond before the creation of the carbon *pseudoradical* centre demanded for the subsequent C–C bond formation, present high activation energies towards ethylene **3** (14.3 kcal·mol^−1^ for the reaction of Ni **1c** with ethylene **3**), *pseudodiradical* TACs such as AY **1a**, which already has the two required carbon *pseudoradical* centres in the structure, present unappreciable activation energies (1.0 kcal·mol^−1^ for the reaction of AY **1a** with ethylene **3**). Note that AY **1a** and Ni **1c** are located at the extremes of the series given in Figure 10.

**Figure 11:**
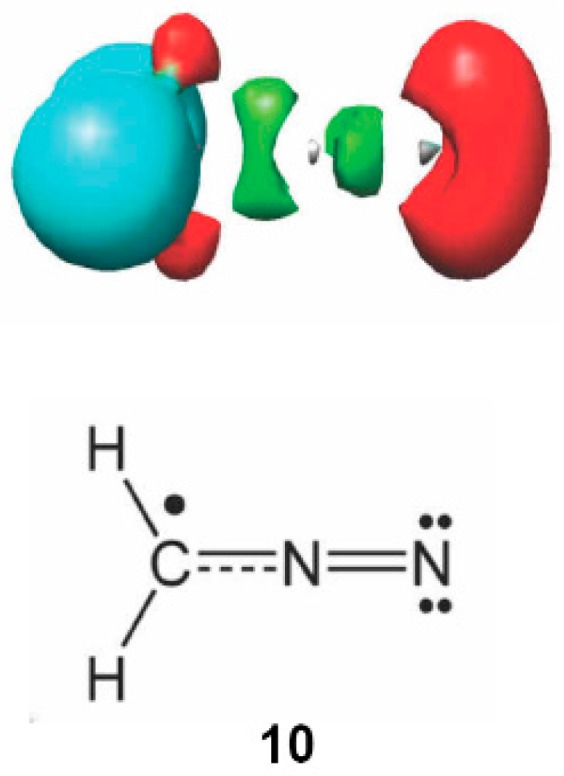
Representation of the ELF localisation domains and the proposed Lewis structure for DAA **2c**.

Very recently, the 32CA reactions of diazoalkanes (DAAs) with ED ethylenes have been studied [36]. ELF topological analysis of the simplest DAA **2c** showed that, similar to AI **1b**, this TAC also has a *pseudoradical* structure (see Figure 11) [36]. However, despite its *pseudoradical* structure, the 32CA reaction of DAA **2c** with ethylene **3** presents a high activation energy, 16.6 kcal·mol^−1^, as a consequence of its lineal P-TAC structure [19].

**Figure 12:**
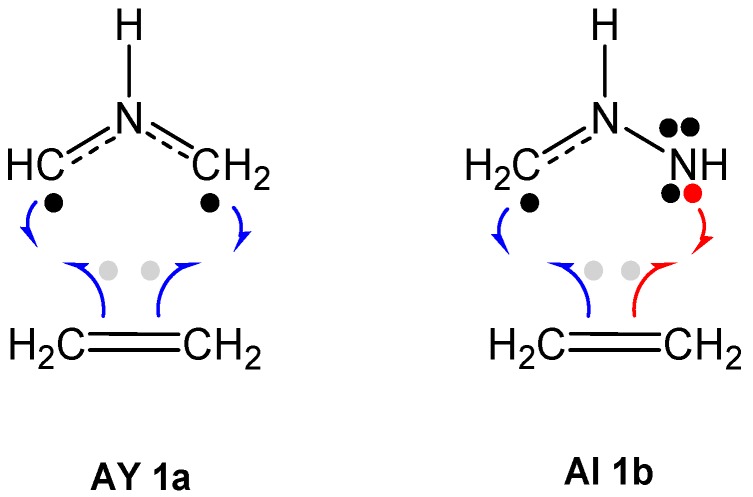
Bonding changes demanded for the C–C bond formation in the *pdr-type* 32CA reaction between AY **1a** and ethylene **3**, and for the C–C and C–N bond formation in *pmr-type* 32CA reaction between AI **1b** and ethylene **3**.

Why AI **1b** having one *pseudoradical* centre presents a different reactivity than AY **1a** having a *pseudodiradical* structure? Figure 12 shows how the two *pseudoradical* centres present in the simplest AY **1a** favour the synchronous C–C single bond formation process through an homolytic rupture of the C–C double bond of the ethylene framework [14], a behaviour that is not feasible in the 32CA reactions of *pseudoradical* AI **1b** and DAA **2c**. Note that, in these TACs, the non-bonding electron density present at the nitrogen atom is associated to the nitrogen sp^2^ lone pairs, which must be redistributed before the creation of the nitrogen *pseudoradical* centre demanded for the C–N single bond formation.

**Scheme 8:**
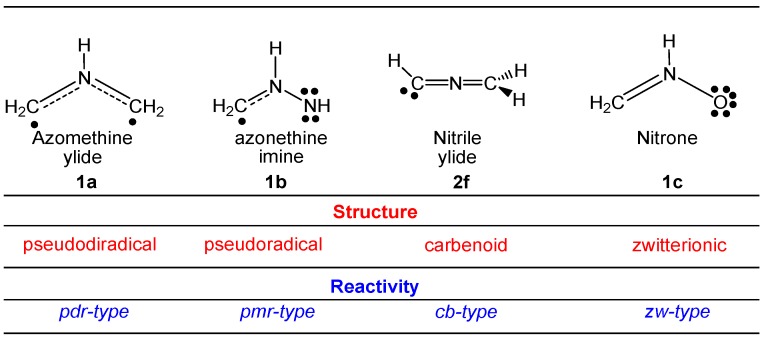
Electronic structure of TACs and the proposed reactivity types in the non-polar 32CA reactions towards ethylene **3**.

Consequently, from the comparison of the electronic structure and the reactivity of AI **1b** in the non-polar 32CA reaction with ethylene **3** with those of the three TACs given in Scheme 3, a new type of 32CA reaction model should be considered. This new type, called *pseudo(mono)radical* type (*pmr-type*) 32CA reaction, is associated with TACs such as AI **1b** and DAA **2c** having only one *pseudoradical* carbon centre, i.e., a *pseudoradical* electronic structure (see Scheme 8). In order to clearly differentiate the reactivity of *pseudodiradical* species from *pseudoradical* ones, we propose to change the original name of *pr-type* (see Scheme 3) to *pdr-type* (see Scheme 8).

Thus, unlike symmetric *pseudodiradical* TACs such as AY **1a**, carbonyl ylides and thiocarbonyl ylides, which induce a symmetric depopulation of the C–C double bond of ethylene **3**, non-symmetric *pseudoradical* TACs such as AIs **1b** and DAAs **2c** are not able to induce an effective symmetric electron density depopulation of the C–C bonding region in the ethylene framework since the electron density demanded for the formation of the new C–N single bonds is at first being part of the non-bonding electron density of the nitrogen sp^2^ lone pairs.

The present theoretical study emphasises how MEDT studies are able to rationalise the reactivity of organic compounds based on a rigorous analysis of the changes of the electron density along organic reactions [12]. Conversely, Houk’s DIEM only permits to establish a good relationship between the *distortion* energy and the activation energy in the series of non-polar 32CA reactions given in Scheme 2, i.e., when more distorted the TS is with respect to the separated reagents, higher the activation energy. This finding, which is a computational assertion of Hammond’s postulate [9], does not resolve the question *why the activation energies of these 32CA reactions depend on the geometry deformations* [7,8].

The molecular geometry is the result of the energy minimisation, which, within the DFT, is a functional of the electron density [10]. Consequently, activation energies, which are the difference in energies between the GS and the TS, should be understood as the energy involved in the changes in electron density demanded to reach the TS. Since the electron density in a molecule and its associated energy depends on the total electrons and the external potential, i.e., the nuclei positions [10], the geometry of any species involved in a reaction path cannot be divided into separated fragments because they lose the information of the whole molecular system.

On the other hand, we have proposed that the GEDT taking place at the TSs is one of the more relevant factors controlling activation energies; the larger GEDT, the lower the activation energy [34]. Such as the non-polar DA reaction between butadiene and ethylene **3** that has a negligible GEDT, the 32CA reactions towards ethylene **3** also do not have any polar character. Consequently, the activation energies implied in these non-polar 32CA reactions are mainly associated to the energy required for the changes in the GS electron density, and not to the geometry deformation such as Houk proposed [7,8], which is a consequence of the former.

## 3. Conclusions

The electronic structure and chemical properties of the simplest AI **1b** as well as its participation in 32CA reactions towards ethylene **3** and ED DCE **6** have been analysed within MEDT using DFT calculations at the MPWB1K/6-311G(d) level.

Analysis of the electron density pattern of the simplest AI **1b** reveals that this TAC presents a *pseudoradical* structure, characterised by the presence of a V(C1) monosynaptic basin integrating 0.62 e at the C1 carbon atom. The charge distribution at AI **1b** does not permit to assign any zwitterionic structure for this TAC, ruling out the common representation of this TAC as a 1,2-dipole.

CDFT analysis of AI **1b** indicates that this TAC is a moderate electrophile and a good nucleophile. Consequently, it is expected that AI **1b** will participate in polar 32CA reactions only towards electrophilically activated ethylenes. Analysis of the nucleophilic $P_{k}^{-}$ indicates that the N3 nitrogen atom is more nucleophilically activated than the C1 carbon.

The 32CA reaction of AI **1b** with ethylene **3** takes place through a one-step mechanism with moderate activation energy, 8.7 kcal·mol^−1^, the reaction being strongly exothermic, −62.0 kcal·mol^−1^. Analysis of the TS geometry shows a high symmetry in the lengths of the two C–C and C–N forming bonds. The GEDT at **TS1**, 0.10 e, indicates that this reaction has a low polar character.

BET analysis of this non-polar 32CA reaction indicates that in spite of the geometrical symmetry found at **TS1**, this one-step mechanism takes place through a non-concerted mechanism in which the C-C bond formation is clearly anticipated prior to the C–N one. Formation of the first C–C single bond takes place at a distance of 2.03 Å through the C-to-C coupling of two carbon *pseudoradical* centres [25], while formation of the second N–C single bond takes place at a distance of 1.92 Å through the C-to-N coupling of two carbon and nitrogen *pseudoradical* centres. Consequently, at **TS1** the formation of the two single bonds has not yet begun.

BET analysis allows the molecular mechanism of the non-polar 32CA reaction to be characterised as a [2n + 2τ] process. This MEDT study makes it possible to reject the concept of *pericyclic mechanism*, since the bonding changes are neither concerted nor do they take place in a cyclic movement. In addition, ELF topological analysis of the structures involved in the formation of the two C–C and C–N single bonds indicates that the C1 *pseudoradical* and N3 non-bonding electron density belonging to AI **1b** and that belonging to the τ bond of ethylene **3** participate in the reaction. This behaviour, which indicates that only two electrons of the TAC and two electrons of the ethylene participate in this reaction, allows rejecting the classification of these reactions as [4π + 2π] processes, and, consequently, the Woodward–Hoffmann rules of the conservation of orbital symmetry [37], according to which this thermal 32CA reactions should be forbidden by the MO symmetry.

The polar 32CA reaction of AI **1b** with DCE **6** also takes place through a one-step mechanism. However, the electrophilic activation of ethylene **3** provokes some remarkable changes in the 32CA reactions of AI **1b** towards ED ethylenes. Now, due to the non-symmetry of both reagents, two regioisomeric channels are feasible. **TS2-m**, associated with the initial C–N bond formation, is found 2.8 kcal·mol^−1^ below **TS2-o**, associated with the initial C–C bond formation; this polar 32CA reaction is highly regioselective, in clear agreement with the CDFT analysis of the Parr functions at the GS of the reagents. The activation barrier of the more favourable **TS2-m** is 8.3 kcal·mol^−1^ lower than that of **TS1**, associated with the non-polar 32CA reaction of AI **1b** with ethylene **3**. This large acceleration is in complete agreement with the high GEDT found at the polar **TS2-m**, 0.25 e, which provokes an electronic stabilisation of the both the nucleophilic and the electrophilic frameworks at the TSs. These behaviours are the consequence of the high nucleophilic character of AI **1b** and the high electrophilic character of DCE **6**.

Interestingly, ELF analysis of the bonding changes along the two regioisomeric channels indicates that the electrophilic activation of the ethylene compound does not only accelerate the reaction, but also changes the mechanism; the non-polar 32CA reaction begins with the C–C single bond formation, while the more favourable channel associated with the polar 32CA reaction starts with the C–N bond formation due to the favourable two-centre interaction between the most nucleophilic centre of AI **1b** and the most electrophilic centre of DCE **6** taking place in a polar process. In addition, while the non-polar reaction is only slightly asynchronous, the polar process takes place through a non-concerted *two-stage one-step* mechanism [33].

Analysis of the electronic structure of AI **1b** and its reactivity towards ethylene **3** indicates that this TAC is different to those previously studied. The *pseudoradical* structure and reactivity of the simplest AI **1b** towards ethylene **3**, such as those of the simplest DAA **10** [36], which are different to the *pseudodiradical* structure and *pdr-type* reactivity of AY **1a**, make it possible to extend Domingo’s classification to a new type of *pseudoradical* TACs and *pmr-type* reactivity.

The present theoretical study emphasises how MEDT is able to rationalise the reactivity of the organic compounds based on a rigorous analysis of the changes of the electron density along organic reactions, thus rejecting obsolete concepts and models developed in the last century through the analysis of MOs [12,25].

## 4. Computational Methods

DFT calculations were performed using the MPWB1K functional [38] together with the 6–311G(d,p) basis set [39]. Optimisations were carried out using the Berny analytical gradient optimisation method [40,41]. The stationary points were characterised by frequency computations in order to verify that TSs have one and only one imaginary frequency. The IRC paths [42] were traced in order to check the energy profiles connecting each TS to the two associated minima of the proposed mechanism using the second order González-Schlegel integration method [43,44]. GEDT [25] is computed by the sum of the natural atomic charges (q), obtained by NPA [45,46], of the atoms belonging to each framework (f) at the TSs; GEDT = Σq_f_. The sign indicates the direction of the electron density flux in such a manner that positive values mean a flux from the considered framework to the other one. All computations were carried out with the Gaussian 09 suite of programs [47].

CDFT [23,24] provides different indices to rationalise and understand chemical structure and reactivity. The global electrophilicity index [48] ω, is given by the following expression, ω = (µ^2^/2η), in terms of the electronic chemical potential µ and the chemical hardness η. Both quantities may be approached in terms of the one-electron energies of the frontier MOs HOMO and LUMO, ε_H_ and ε_L_, as µ ≈ (ε_H_ + ε_L_)/2 and η ≈ (ε_L_ − ε_H_), respectively [49,50]. The global nucleophilicity index [51,52], *N*, based on the HOMO energies obtained within the Kohn-Sham scheme [53], is defined as *N* = E_HOMO_(Nu) − E_HOMO_(TCE), where tetracyanoethylene (TCE) is the reference. The *pr* index, which has recently been introduced in order to characterise the participation of *pseudodiradical* TACs in *pdr-type* 32CA reactions [16], comprises the chemical hardness η and the nucleophilicity *N* index of the TAC, as *pr* = *N*/η. Electrophilic *P_k_^-^* and nucleophilic *P_k_^-^* Parr functions [28] were obtained through the analysis of the Mulliken atomic spin densities (ASD) of the radical anion of DCE **6** and the radical cation of AI **1b** by single point calculations from the neutral species. DFT reactivity indices were computed at the B3LYP/6-31G(d) level.

ELF [54] studies were performed with the TopMod [55] program and using the corresponding B3LYP/6-311G(d,p) monodeterminantal wavefunctions over a grid spacing of 0.1 a.u.. For the BET study, the corresponding reaction channel was followed by performing the topological analysis of the ELF for 862 nuclear configurations along the IRC path. A BET procedure was used for the characterisation of the bond formation processes along the two *meta/ortho* regioisomeric channels associated to the polar reaction by performing the topological analysis of the ELF for 198 (*meta*) and 472 (*ortho*) nuclear configurations.

## Figures and Tables

**Table 1:** B3LYP/6-31G(d) electronic chemical potential, μ, chemical hardness, η, electrophilicity, ω, and nucleophilicity, *N*, in eV, for A-TACs **1a**–**c**, ethylene **3** and DCE **6**, and the *pr* index of **1a**–**c**.

	µ	η	ω	*N*	*pr*
AY **1a**	−1.82	4.47	0.37	5.07	1.13
AI **1b**	−2.70	5.02	0.72	3.92	0.78
Ni **1c**	−3.43	5.55	1.06	2.92	0.53
Ethylene **3**	−3.37	7.77	0.73	1.86	
DCE **6**	−5.64	5.64	2.82	0.65	

## Supplementary Materials

Supplementary materials are available online. BET study of the non-polar 32CA reaction between AI **1b** and ethylene **3**. ELF topological analysis of the C–C and N–C bond formation processes along the *meta* and *ortho* regioisomeric channel associated with the polar 32CA reaction between AI **1b** and DCE **6**.

## Abbreviations

The following abbreviations are used in this manuscript:

32CA	[3+2] cycloaddition
AI	azomethine imine
ASD	atomic spin densities
A-TAC	allylic-type TAC
BET	Bonding Evolution Theory
*cb-type*	carbenoid-type
CDFT	Conceptual DFT
DAA	diazoalkanes
DCE	dicyanoethylene
DFT	Density Functional Theory
DIEM	Distortion/Interaction Energy Model
ED	electron-deficient
ELF	electron localisation function
FMO	Frontier Molecular Orbital
GEDT	global electron density transfer
GS	ground state
MC	molecular complex
MEDT	Molecular Electron Density Theory
MO	molecular orbital
Ni	nitrone
*pdr-type*	*pseudodiradical*-type
*pmr-type*	*pseudoradical*-type
P-TAC	propargylic-type TAC
TAC	three-atom-component
TCE	tetracyanoethylene
TS	transition state structure
*zw-type*	zwitterionic-type
