# Supplementary material for: A Molecular Electron Density Theory Study of the Reactivity of Azomethine Imine in [3+2] Cycloaddition Reactions
Source: Molecules. 2017 May 6;22(5):750. doi: 10.3390/molecules22050750 (PMC6154604; doi:10.3390/molecules22050750)
Supplement: Supplementary file 1 [file molecules-22-00750-s001.pdf]

# Supplementary Material

## A Molecular Electron Density Theory Study of the Reactivity of Azomethine Imine in [3+2] Cycloaddition Reactions

Luis R. Domingo and Mar Ríos-Gutiérrez

Department of Organic Chemistry, University of Valencia, Dr. Moliner 50, E-46100 Burjassot, Valencia, Spain
e-mail: domingo@utopia.uv.es
web: www.luisrdomingo.com

### Index

**S2** **Table S1.** MPWB1K/6-311G(d) total electronic energies of the stationary points involved in the 32CA reaction between AI **1b** and ethylene **3**.

**S3** **Table S2.** MPWB1K/6-311G(d) total electronic energies of the stationary points involved in the 32CA reaction between AI **1b** and DCE **6**.

**S4** 1. Bonding Evolution Theory (BET) study of the non-polar 32CA reaction between AI **1b** and ethylene **3**

**S12** 2. Electron localisation function (ELF) topological analysis of the C–C and N–C bond formation processes along the polar 32CA reaction between AI **1b** and DCE **6**

**S12** 2.1. ELF topological analysis along the more favourable *meta* regioisomeric channel

**S17** 2.2. ELF topological analysis along the less favourable *ortho* regioisomeric channel

**S22** 3. Theoretical background

**S22** 3.1. Topological analysis of the ELF

**S23** 3.2. BET

**S25** 4. References

**Table S1.** MPWB1K/6-311G(d) total electronic energies, in a.u., of the stationary points involved in the 32CA reaction between AI **1b** and ethylene **3**.

| | E |
|---|---|
| AI **1b** | -149.866583 |
| ethylene **3** | -78.546281 |
| **MC1** | -228.416728 |
| **TS1** | -228.404441 |
| **5** | -228.511604 |

**Table S2.** MPWB1K/6-311G(d) total electronic energies, in a.u., of the stationary points involved in the 32CA reactions between AI **1b** and DCE **6**.

| | E |
|---|---|
| AI **1b** | -149.866583 |
| DCE **6** | -262.985589 |
| **MC2-o** | -412.862154 |
| **MC2-m** | -412.866330 |
| **TS2-o** | -412.861212 |
| **TS2-m** | -412.865718 |
| **7** | -412.941788 |
| **8** | -412.944843 |

## 1. Bonding Evolution Theory (BET) study of the non-polar 32CA reaction between AI 1b and ethylene 3

The BET study of the non-polar 32CA reaction between AI **1b** and ethylene **3** indicates that this reaction can be topologically characterised by nine differentiated phases. Simplified representation of the molecular mechanism of the non-polar 32CA reaction between AI **1b** and ethylene **3** by ELF-based Lewis structures is shown in Scheme S1; the nine phases, defined by the points **Pi**, in which the intrinsic reaction coordinate (IRC) is topologically divided are shown in Figure S1 by black dashed lines crossing the energy curve at the corresponding points; the populations of the most significant valence basins (those associated with the bonding regions directly involved in the reaction) of the selected points of the IRC, **Pi**, are included in Table S3; the attractor positions of the electron localisation function (ELF) basins for the points involved in the bond formation processes are shown in Figure S2 and the basin-population changes along the reaction path are graphically represented in Figure S3.

The long *Phase I* (see Figure S1), 3.718 Å ≥ d(C1–C5) > 2.908 Å and 3.337 Å ≥ d(N3–C4) > 2.800 Å, begins at molecular complex (MC) **MC1**, which is a minimum in the reaction path connecting the separated reagents, AI **1b** and ethylene **3**, with the TS of the reaction, **TS1**. The bonding pattern of a MC usually resembles that of the separated reagents. Thus, ELF topological analysis of **MC1** only reveals slight changes in the ELF valence basin electron populations in the AI and ethylene frameworks with respect to the topological features of AI **1b** (see Section 3.1) and ethylene **3** (see Table S3). The two V(C1) and V'(C1) monosynaptic basins already present in AI **1b**, which are associated with a C1 *pseudoradical* center and which characterise its *pseudoradical* electronic structure, are also observed at **MC1** integrating a total population of 0.57 e. It should be emphasised that along this phase, the V'(C1) monosynaptic basin oriented above the molecular plane of the AI framework decreases its electron population from 0.29 e to 0.08 e at the end of this phase, its electron density being redistributed among the adjacent hydrogen atoms. On the other hand, the two V(C4,C5) and V'(C4,C5) disynaptic basins in the ethylene moiety, integrating 1.70 e and 1.69 e, suggest the presence of a C4–C5 double bond according to the Lewis chemical bond model. At **MC1**, the global electron density transfer [1] (GEDT) is negligible, 0.01 e.

*Phase II*, which is also long (see Figure S1), 2.908 Å ≥ d(C1–C5) > 2.311 Å and 2.800 Å ≥ d(N3–C4) > 2.319 Å, begins at **P1**. At this point, the V'(C1) monosynaptic basin present at **MC1** has disappeared by means of a cusp *C* catastrophe, while the V(C1) monosynaptic basin remains with a population of 0.25 e. This topological change can be related to a rehydridisation process of the hybridised $sp^2$ C1 carbon of the AI framework to the $sp^3$ hybridisation demanded for the formation of the C1–C5 single bond. At **P1**, the GEDT has slightly increased to 0.04 e but remains very low.

The changes in electron density taking place along *Phases I* and *II*, which are mainly related to the rehydridisation process of the C1 carbon from $sp^2$ to $sp^3$ and to the increase of the population of the C1–N2 bonding region, imply an energy cost (EC) of *ca.* 7.6 kcal·mol$^{-1}$ (see Table S3).

The short *Phase III* (see Figure S1), 2.311 Å ≥ d(C1–C5) > 2.297 Å and 2.319 Å ≥ d(N3–C4) > 2.307 Å, begins at **P2**. At this point, which corresponds to a second cusp *C* catastrophe, the two V(C4,C5) and V'(C4,C5) disynaptic basins present at the ethylene framework of the previous **P1** have merged into a new V(C4,C5) disynaptic basin integrating 3.20 e, after having been depopulated by a total of 0.17 e along the previous *Phase II*. At the AI framework, while the population of the V(N3) monosynaptic and the V(N2,N3) disynaptic basins has decreased to 3.32 e and 1.90 e, that of the V(C1,N2) disynaptic basin has increased to 3.31 e. In addition, the V(C1) monosynaptic basin has doubled its population, 0.50 e, recovering part of the electron density previously redistributed among the hydrogen atoms. At **P2**, the GEDT has reached the maximum value along the reaction, 0.10 e, which is suggestive of the low polar character of this 32CA reaction.

*Phase IV*, 2.297 Å ≥ d(C1–C5) > 2.174 Å and 2.307 Å ≥ d(N3–C4) > 2.206 Å, begins at **P3**. This point is characterised as a fold $F^{\dagger}$ catastrophe implying the creation of a new V(N2) monosynaptic basin at the N2 nitrogen of the AI framework with an initial population of 0.76 e. The electron density of this V(N2) monosynaptic basin, which is associated with the non-bonding N2 lone pair present at the final pyrazolidinone **5**, comes entirely from the sudden strong depopulation of the V(C1,N2) disynaptic basin experienced at **P3** to 2.55 e. This behaviour, *i.e.* population of the V(N2) monosynaptic basin at the expense of the depopulation of the V(C1,N2) disynaptic basin, will be maintained throughout the reaction. At **P3**, the GEDT remains 0.10 e. At this phase, the TS of the reaction, **TS1**, d(C1–C5) = 2.272 Å and d(N3–C4) =

2.286 Å, is found. Only slight changes in electron density at the TS with respect to **P3** are observed.

The changes in electron density taking place along *Phases III* and *IV*, which are mainly related to the depopulation of the C1–N2 and C4–C5 bonding regions, release a slight molecular relaxation energy (MRE) of *ca.* 0.9 kcal·mol$^{-1}$ (see Table S3).

*Phase V*, 2.174 Å ≥ d(C1–C5) > 2.091 Å and 2.206 Å ≥ d(N3–C4) > 2.136 Å, begins at **P4**. This point is characterised as a fold $F^{\dagger}$ catastrophe implying the creation of a new V(C5) monosynaptic basin at the C5 carbon of the ethylene framework with an initial population of 0.25 e. The electron density of this V(C5) monosynaptic basin, which is demanded for the C1–C5 single bond formation, comes entirely from the sudden strong depopulation of the V(C4,C5) disynaptic basin experienced at **P4** to 2.96 e. At **P4**, at which the GEDT has slightly decreased to 0.09 e, the GEDT begins a progressive decrease until the reaction is finished.

The very short *Phase VI* (see Figure S1), 2.091 Å ≥ d(C1–C5) > 2.033 Å and 2.136 Å ≥ d(N3–C4) > 2.086 Å, begins at **P5**. This point is characterised as a fold $F^{\dagger}$ catastrophe implying the creation of a new V(C4) monosynaptic basin at the C4 of the ethylene framework with an initial population of 0.21 e. Such as in the creation of the V(C5) monosynaptic basin, the electron density of the V(C4) monosynaptic basin, which is demanded for the N3–C4 single bond formation, comes entirely from the sudden strong depopulation of the V(C4,C5) disynaptic basin experienced at **P5** to 2.63 e. At **P5**, the V(C5) monosynaptic basin has reached an electron density of 0.40 e. It is interesting to note that the three C1, C4 and C5 *pseudoradical* centers required for the formation of the new C1–C5 and N3–C4 are simultaneously present at **P5** (see **P5** in Figure S2).

*Phase VII*, 2.033 Å ≥ d(C1–C5) > 1.868 Å and 2.086 Å ≥ d(N3–C4) > 1.937 Å, begins at **P6** by means of a cusp *C* catastrophe. In this phase, the first most relevant change along the IRC take places; the two V(C1) and V(C5) monosynaptic basins present in the previous *Phase VI* have merged into a new V(C1,C5) disynaptic basin with an initial population of 1.19 e (see **P6** in Figure S2 and the merger of V(C1) and V(C5), in green at **P5**, into V(C1,C5), in blue at **P6**, in Figure S3). This noteworthy topological change indicates that the formation of the first C1–C5 single bond begins at a C–C distance of *ca.* 2.03 Å, through the C-to-C coupling of the two C1 and C5

*pseudoradical* centers [1]. Note that the C1 *pseudoradical* center was already present at AI **1b** (see Table S3).

*Phase VIII*, 1.868 Å ≥ d(C1–C5) > 1.849 Å and 1.937 Å ≥ d(N3–C4) > 1.919 Å, begins at **P7** by means of a cusp $C^{\dagger}$ catastrophe and is also very short (see Figure S1). The most relevant topological change observed at **P7** is the split of the V(N3) monosynaptic basin present at **P6** into two new monosynaptic basins, V(N3) and V'(N3), integrating 2.67 e and 0.58 e, respectively (see **P7** in Figure S2). Interestingly, the new V'(N3) monosynaptic basin, which is demanded for the subsequent N3–C4 single bond formation, allows characterising the N3 nitrogen as a *pseudoradical* center.

Finally, the last and long *Phase IX* (see Figure S1), 1.849 Å ≥ d(C1–C5) ≥ 1.525 Å and 1.919 Å ≥ d(N3–C4) ≥ 1.463 Å, begins at **P8** by means of a cusp *C* catastrophe and ends at pyrazolidinone **5**. In this phase, the second most relevant change along the IRC takes place. The two V'(N3) and V(C4) monosynaptic basins present at **P7** have merged into a new V(N3,C4) disynaptic basin with an initial population of 1.03 e (see **P8** in Figure S2 and the merger of V'(N3) and V(C4), in green at **P7**, into V(N3,C4), in blue at **P8**, in Figure S3). This noteworthy topological change indicates that the formation of the second N3–C4 single bond begins at an N–C distance of *ca.* 1.92 Å, by a C-to-N coupling of the two N3 and C4 *pseudoradical* centers.

At pyrazolidinone **5**, no significant topological changes with respect to the topological features of the last **P8** are observed. Along this phase, the two disynaptic basins, V(C1,C5) and V(N3,C4), associated with the new single bonds have reached an electron density of 1.74 e and 1.88 e, respectively. Interestingly, the electron density gathered in the N3–C4 bonding region along *Phase IX* mainly comes from the V(N3) monosynaptic basin, which reaches an electron density of 2.28 e at pyrazolidinone **5**.

From this BET study, the molecular mechanism of the non-polar 32CA reaction between the simplest AI **1b** and ethylene **3** can be summarised as follows (see Scheme S1): i) the reaction begins with the disappearance of the V'(C1) monosynaptic basin associated with the hybridised $sp^2$ C1 *pseudoradical* center of AI **1b** (*Phases I* and *II*) in order to achieve the $sp^3$ hibridisation demanded for the formation of the C1–C5 single bond. This rehybridisation process entails a moderate EC of 7.6 kcal·mol$^{-1}$; ii) then, the V(C1,N2), and the V(C4,C5) and V'(C4,C5) disynaptic basins related to the C1–N2 and C4–C5 double bond regions of the AI and ethylene frameworks begin to depopulate

releasing a slight MRE of 0.9 kcal·mol$^{-1}$ along *Phases III* and *IV*, and provoking the appearance of the V(N2) monosynaptic basin associated with the non-bonding N2 lone pair present at the final pyrazolidinone **5**; iii) this depopulation permits the creation of two V(C4) and V(C5) monosynaptic basins at the ethylene framework (*Phases VI* and *V*), which are related to the C4 and C5 *pseudoradical* centers demanded for the formation of the new C1–C5 and N3–C4 single bonds; iv) formation of the V(C1,C5) disyanaptic basin associated with the first new C1–C5 single bond takes place at a C–C distance of 2.03 Å through the merger of the two V(C1) and V(C5) monosynaptic basins (*Phase VII*) [1]; v) then, the electron density around the N3 nitrogen is redistributed in such a manner that a new V'(N3) monosynaptic basin related to an N3 *pseudoradical* center is created (*Phase VIII*); and vi) finally, formation of the V(N3,C4) disynaptic basin associated with the second new N3–C4 single bond takes place at the end of the reaction path at an N–C distance of 1.92 Å through the merger of the two V(N3) and V(C4) monosynaptic basins (*Phase IX*) [1].

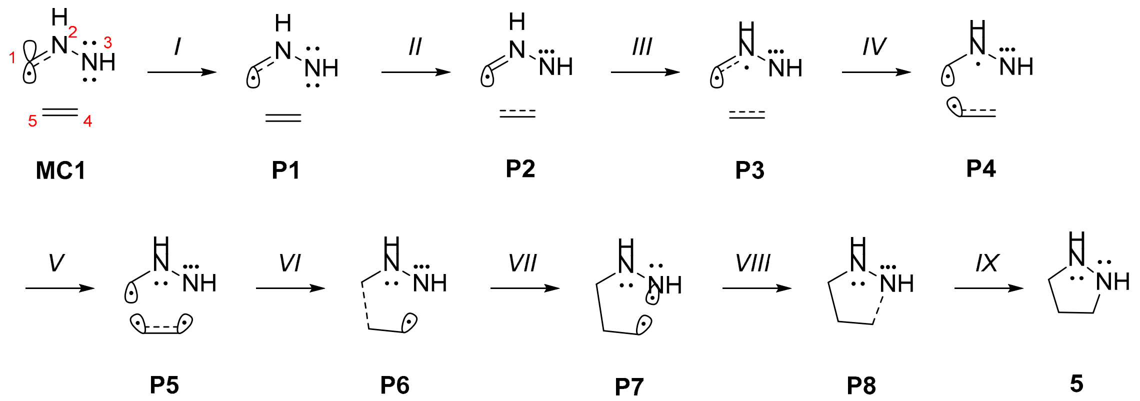


**Scheme S1**. Simplified representation of the molecular mechanism of the non-polar 32CA reaction between AI **1b** and ethylene **3** by ELF-based Lewis structures.

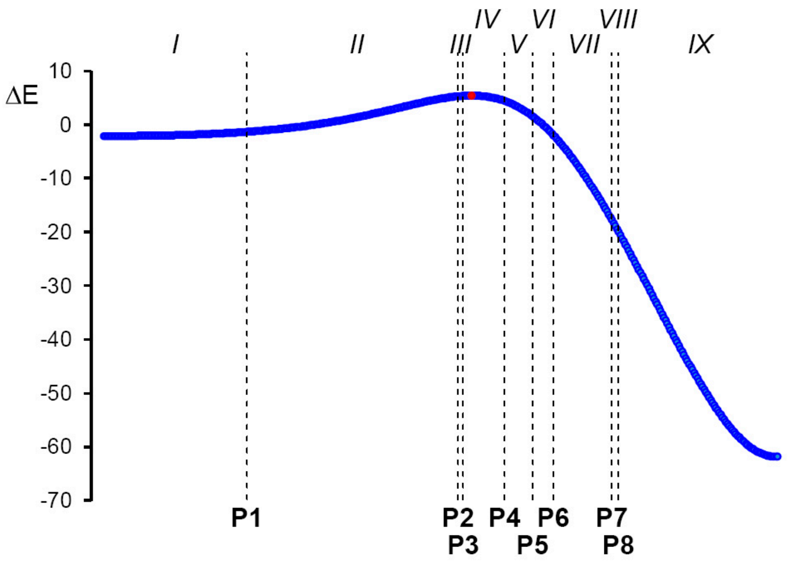


**Figure S1**. The nine phases, defined by the points **Pi** and separated by black hyphened lines, in which the IRC (in amu$^{1/2}$·Bohr) associated with the non-polar 32CA reaction between AI **1b** and ethylene **3** is topologically divided, separated by black hyphened lines crossing the energy curve at the points **Pi**. Relative energy variations, ΔE, with respect to the separated reagents, are given in kcal·mol$^{-1}$, while the red point indicates the position of **TS1**.

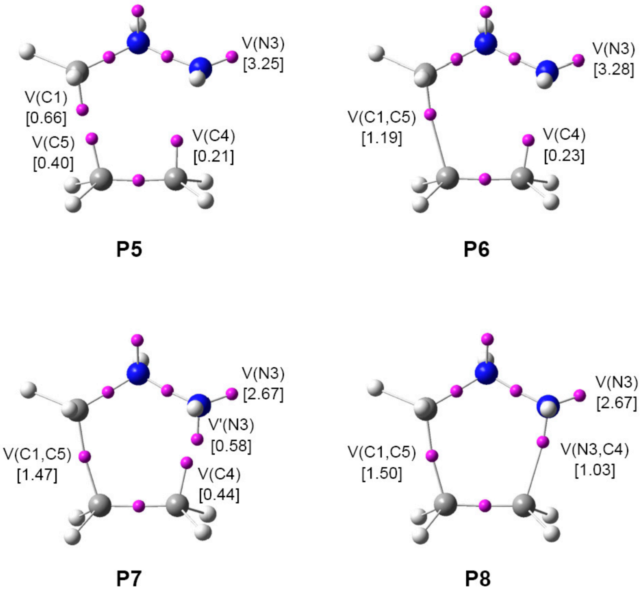


**Figure S2**. ELF attractor positions for the points of the IRC defining *Phases VI – IX* involved in the formation of the C1–C5 and N3–C4 single bonds along the non-polar 32CA reaction between AI **1b** and ethylene **3**. The electron populations, in e, are given in brackets.

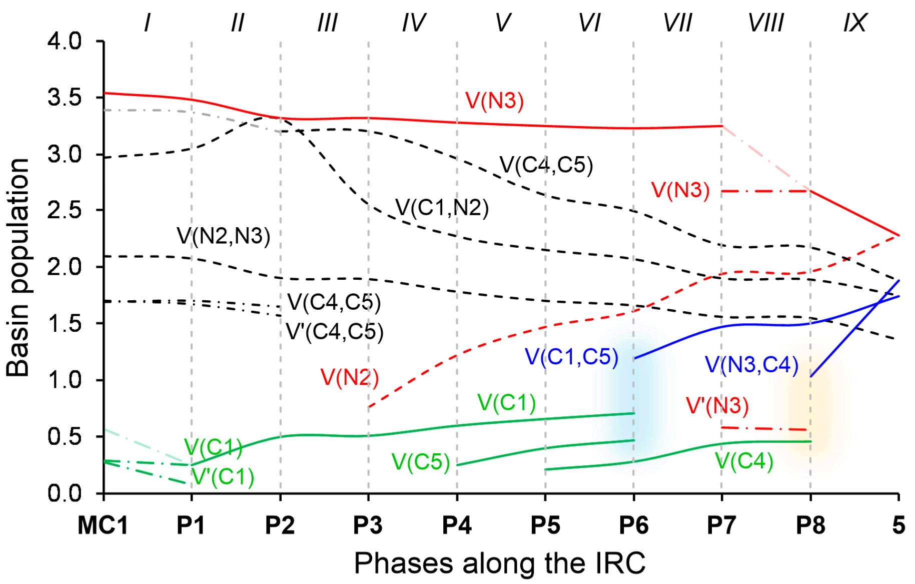


**Figure S3**. Graphic representation of the basin population changes along the non-polar 32CA reaction between AI **1b** and ethylene **3**. Dash dotted curves represent bonding regions described by two basins, dashed curves represent bonding regions described by only one basin and continuous curves represent the basins directly involved in the formation of the new single bonds; black curves represent basins that do not participate in the bond formation processes, grey curves represent the sum of basins characterising a bonding region, the red colour is for lone pairs, green for carbon *pseudoradical* centers and blue for the new formed single bonds.

**Table S3**. Valence basin populations N calculated from the ELF of the IRC points, **P1** – **P8**, defining the nine phases characterising the molecular mechanism of the non-polar 32CA reaction between AI **1b** and ethylene **3**. The stationary points **MC1**, **TS1** and **5** are also included. Distances are given in Å, GEDT values and electron populations in e, and relative energies in kcal·mol$^{-1}$.

| Points | **1b** | **3** | **MC1** | **P1** | **P2** | **P3** | **P4** | **P5** | **P6** | **P7** | **P8** | **5** | **TS1** |
|---|---|---|---|---|---|---|---|---|---|---|---|---|---|
| Catastrophes | | | | *C* | *C* | *F*† | *F*† | *F*† | *C* | *C*† | *C* | | |
| Phases | | | | *I* | *II* | *III* | *IV* | *V* | *VI* | *VII* | *VIII* | *IX* | |
| d(C1–C5) | | | 3.718 | 2.908 | 2.311 | 2.297 | 2.174 | 2.091 | 2.033 | 1.868 | 1.849 | 1.525 | 2.272 |
| d(N3–C4) | | | 3.337 | 2.800 | 2.319 | 2.307 | 2.206 | 2.136 | 2.086 | 1.937 | 1.919 | 1.463 | 2.286 |
| DE | | | -2.4 | -1.5 | 5.2 | 5.2 | 4.3 | 1.3 | -2.1 | -17.9 | -20.1 | -62.0 | 5.3 |
| GEDT | | | 0.01 | 0.04 | 0.10 | 0.10 | 0.09 | 0.07 | 0.05 | -0.02 | -0.03 | -0.13 | 0.10 |
| V(C1,N2) | 2.95 | | 2.97 | 3.05 | 3.31 | 2.55 | 2.27 | 2.15 | 2.07 | 1.90 | 1.89 | 1.75 | 2.51 |
| V(N2) | | | | | | 0.76 | 1.22 | 1.47 | 1.61 | 1.94 | 1.96 | 2.28 | 0.84 |
| V(N2,N3) | 2.10 | | 2.09 | 2.07 | 1.90 | 1.89 | 1.78 | 1.70 | 1.66 | 1.56 | 1.55 | 1.36 | 1.87 |
| V(C4,C5) | | 1.71 | 1.70 | 1.67 | 3.20 | 3.20 | 2.96 | 2.63 | 2.49 | 2.19 | 2.17 | 1.88 | 3.18 |
| V'(C4,C5) | | 1.71 | 1.69 | 1.70 | | | | | | | | | |
| V(C1) | 0.31 | | 0.28 | 0.25 | 0.50 | 0.51 | 0.60 | 0.66 | | | | | 0.52 |
| V'(C1) | 0.31 | | 0.29 | | | | | | | | | | |
| V(N3) | 3.53 | | 3.54 | 3.48 | 3.32 | 3.32 | 3.28 | 3.25 | 3.23 | 2.67 | 2.67 | 2.28 | 3.32 |
| V'(N3) | | | | | | | | | | 0.58 | | | |
| V(C4) | | | | | | | | 0.21 | 0.28 | 0.44 | | | |
| V(C5) | | | | | | | 0.25 | 0.40 | | | | | |
| V(C1,C5) | | | | | | | | | 1.19 | 1.47 | 1.50 | 1.74 | |
| V(N3,C4) | | | | | | | | | | | 1.03 | 1.88 | |

## 2. ELF topological analysis of the C−C and N−C bond formation processes along the polar 32CA reaction between AI 1b and DCE 6

*2.1 ELF topological analysis along the more favourable meta regioisomeric channel*

The populations of the most significant valence basins of the stationary points and of the selected points of the IRC involved in the formation of the new C1−C5 and N3−C4 single bonds are included in Table S4, while the attractor positions of the ELF basins for the points involved in the bond formation processes are shown in Figure S4.

At **MC2-m**, d(C1−C5) = 2.957 Å and d(N3−C4) = 2.462 Å, the AI framework appears characterised by two disynaptic basins, V(C1,N2) and V(N2,N3), integrating 3.39 e and 2.02 e, and one V(N3) monosynaptic basin with a population of 3.46 e. According to the Lewis chemical bond model, these two disynaptic basins could be associated to C1−N2 double and N2−N3 single bonds, while the monosynaptic basin could be related to the non-bonding N3 electron density commonly represented as two N3 lone pairs. On the other hand, two V(C4,C5) and V'(C4,C5) disynaptic basins describe the C4−C5 double bond of the DCE moiety integrating a total population of 3.29 e. Although the bonding pattern of MCs usually resembles that of the separated reagents, see **MC1**, in this case the ELF picture of **MC2-m** is somewhat different. Note that the two V(C1) and V'(C1) monosynaptic basins characterising the C1 *pseudoradical* center of AI **1b** are not present at **MC2-m**, while the double bond character of the C1−N2 bonding region has increased. At **MC2-m**, the GEDT is already high, 0.17 e.

At **TS2-m**, d(C1−C5) = 2.796 Å and d(N3−C4) = 2.142 Å, the slight depopulation of the two V(C4,C5) and V'(C4,C5) disynaptic basins of the DCE framework by only 0.03 e have provoked their merger into one single V(C4,C5) disynaptic basin integrating 3.26 e. Meanwhile, at the AI framework, only a slight electron density redistribution can be noticed; while the population of the V(C1,N2) disynaptic basin increases to 3.46 e, the V(N2,N3) disynaptic and V(N3) monosynaptic basins are depopulated to 1.89 e and 3.40 e At **TS2-m**, the GEDT has increased to 0.27 e, a high value for 32CA reactions that allows establishing the high polar character of the 32CA reaction between AI **1b** and DCE **6**.

The changes in electron density taking place coming from **MC2-m** to **TS2-m**, which are very scarce and only imply slight electron density variations within the

molecular system mainly directed towards the rupture of the C4–C5 double bond of DCE **6** and depopulation of the N2–N3 bonding region, account for the very low activation barrier of *ca.* 0.4 kcal·mol$^{-1}$ from **MC2-m** (see Table S4).

At **P1-m**, d(C1–C5) = 2.680 Å and d(N3–C4) = 1.822 Å, a new V(C5) monosynaptic basin, integrating 0.31 e, is observed at the C5 carbon of the ethylene framework. The electron density of this monosynaptic basin, which is related to the C5 *peudoradical* center demanded for the formation of the new C1–C5 single bond, proceeds from the V(C4,C5) disynaptic basin, whose population has strongly decreased to 2.79 e at this point, and from the GEDT taking place along this polar reaction. At **P1-m**, the GEDT has continued increasing to the high value of 0.39 e.

At **P2-m**, d(C1–C5) = 2.675 Å and d(N3–C4) = 1.810 Å, the presence of a new V(N3,C4) disynaptic basin integrating 0.98 e together with the strong depopulation of the V(N3) monosynaptic basin to 2.64 e indicates that the formation of the first N3–C4 single bond begins at an N–C distance of *ca.* 1.81 Å through the donation of part of the non-bonding electron density of the N3 nitrogen to the C4 carbon (see **P1-m** and **P2-m** in Figure S4). Note that the N3 and C4 atoms are the most nucleophilic and electrophilic sites of AI **1b** and DCE **6**, respectively. At **P2-m**, simultaneously to the formation of the first N3–C4 single bond, the maximum GEDT along the reaction path is reached, 0.42 e, which emphasises the strong polar character of this 32CA reaction.

At **P3-m**, d(C1–C5) = 2.318 Å and d(N3–C4) = 1.521 Å, the V(C1) monosynaptic basin associated with the C1 *pseudoradical* center of AI **1b** is again observed with an electron population of 0.15 e as a consequence of the strong depopulation experienced by the V(C1,N2) disynaptic basin to 2.38 e (see **P3-m** in Figure S4). This lost electron density together with the depopulation of the V(N2,N3) disynaptic basin to 1.48 e also contributes to the appearance of a new V(N2) monosynaptic basin, integrating 1.75 e at **P3-m**, related to the non-bonding N2 nitrogen lone pair present at pyrazolidinone **8**. At this point, the V(C5) monosynaptic basin has reached a high electron population of 0.67 e. Note that the two C1 and C5 *pseudoradical* centers demanded for the subsequent C1–C5 bond formation are still present at this point, when the N3–C4 bond formed first has reached *ca.* 91% of its final population at pyrazolidinone **8**. At **P3-m**, the GEDT has decreased to 0.27 e due to a retro-donation process from the DCE to the AI substructures.

At **P4-m**, d(C1−C5) = 2.145 Å and d(N3−C4) = 1.500 Å, while the V(C1) and V(C5) monosynaptic basins have disappeared, a new V(C1,C5) disynaptic basin associated with the formation of the second C1−C5 single bond is observed between the C1 and C5 carbons with an electron population of 1.09 e, thus indicating that the formation of the second C1−C5 single bond starts at a C−C distance of *ca.* 2.14 through the C-to-C coupling of the two C1 and C5 *pseudoradical* centers (see **P4-m** in Figure S4) [1]. The V(N3,C4) disynaptic basin formed first has reached 1.64 e at this point, which is the 95% of its final population at pyrazolidinone **8**, thus characterising a *two-step one-step* mechanism in which the formation of the second bond begins when the formation of the first single bond is practically completed [2]. At **P4-m**, the GEDT is 0.14 e.

Finally, at pyrazolidinone **8**, d(C1−C5) = 1.564 Å and d(N3−C4) = 1.441 Å, the new V(C1,C5) and V(N3,C4) disynaptic basins reach populations of 1.82 e and 1.72 e. Interestingly, while the populations of the V(C4,C5) and V(C1,N2) disynaptic basins are similar to those of the former, *i.e.* 1.83 e and 1.74 e, the V(N2,N3) disynaptic basin has a lower electron population, 1.38 e. At last, the two V(N2) and V(N3) monosynaptic basins related to the two non-bonding N2 and N3 nitrogen lone pairs integrate 2.25 e and 2.24 e. At **8**, the GEDT is negligible, 0.03 e.

**Table S4**. Valence basin populations N calculated from the ELF of the stationary points and some points of the IRC involved in the formation of the new C1–C5 and N3–C4 single bonds along the more favourable *meta* regioisomeric channel associated with the polar 32CA reaction between AI **1b** and DCE **6**. Distances are given in Å, GEDT values and electron populations in e, and relative[a] energies in kcal·mol$^{-1}$.

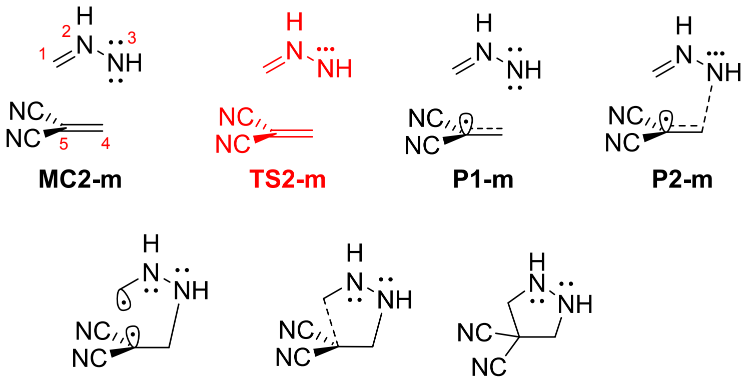

**MC2-m** **TS2-m** **P1-m** **P2-m**

**P3-m** **P4-m** **8**

| Points | **1b** | **6** | **MC2-m** | **TS2-m** | **P1-m** | **P2-m** | **P3-m** | **P4-m** | **8** |
|---|---|---|---|---|---|---|---|---|---|
| d(C1–C5) | | | 2.957 | 2.796 | 2.680 | 2.675 | 2.318 | 2.145 | 1.564 |
| d(N3–C4) | | | 2.462 | 2.142 | 1.822 | 1.810 | 1.521 | 1.500 | 1.441 |
| ΔE | | | -8.9 | -8.5 | -11.1 | -11.4 | -23.1 | -29.5 | -58.2 |
| GEDT | | | 0.17 | 0.27 | 0.39 | 0.40 | 0.34 | 0.26 | 0.03 |
| V(C1,N2) | 2.95 | | 3.39 | 3.46 | 3.64 | 3.67 | 2.38 | 2.19 | 1.74 |
| V(N2) | | | | | | | 1.75 | 1.78 | 2.25 |
| V(N2,N3) | 2.10 | | 2.02 | 1.89 | 1.72 | 1.70 | 1.48 | 1.42 | 1.38 |
| V(C4,C5) | | 1.66 | 1.66 | 3.26 | 2.79 | 2.78 | 2.18 | 2.07 | 1.83 |
| V'(C4,C5) | | 1.66 | 1.63 | | | | | | |
| V(C1) | 0.31 | | | | | | 0.15 | | |
| V'(C1) | 0.31 | | | | | | | | |
| V(N3) | 3.53 | | 3.46 | 3.40 | 3.61 | 2.64 | 2.32 | 2.27 | 2.24 |
| V(C5) | | | | | 0.31 | 0.31 | 0.67 | | |
| V(C1,C5) | | | | | | | | 1.09 | 1.82 |
| V(N3,C4) | | | | | | 0.98 | 1.58 | 1.64 | 1.73 |

[a] Relative to the separated reagents AI **1b** and DCE **6**.

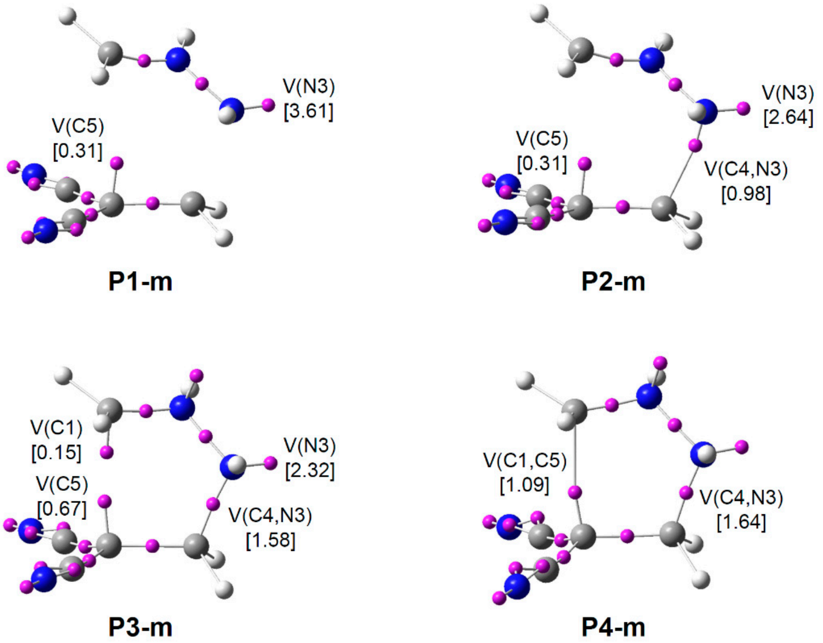


**Figure S4**. ELF attractor positions for the selected points of the IRC involved in the formation of the C1−C4 and N3−C5 single bonds along the more favourable *meta* regioisomeric channel associated with the polar 32CA reaction between AI **1b** and DCE **6**. The electron populations, in e, are given in brackets.

*2.2 ELF topological analysis along the less favourable ortho regioisomeric channel*

The populations of the most significant valence basins of the stationary points and of the selected points of the IRC involved in the formation of the new C1–C4 and N3–C5 single bonds are included in Table S5, while the attractor positions of the ELF basins for the points involved in the bond formation processes are shown in Figure S5.

ELF topological analysis of **MC2-o**, d(C1–C4) = 2.758 Å and d(N3–C5) = 2.752 Å, shows a similar bonding pattern to that of the separated reagents; in the AI framework, besides the two V(C1,N2) and V(N2,N3) disynaptic basins, integrating 2.98 e and 2.12 e, associated to the two C1–N2 and N2–N3 bonding regions, a V(N3) monosynaptic basin, related with the non-bonding N3 electron density, is observed with an electron population of 3.36 e. It should be noted that the immediate loss of the planar arrangement around the C1 carbon at **MC2-o** in order to achieve the non-planar orientation demanded for the C–C bond formation makes that, unlike **1b**, only one V(C1) monosynaptic basin characterises the C1 *pseudoradical* center with approximately half of the electron density gathered at **1b**, 0.38 e. Additionally, the two V(C4,C5) and V’(C4,C5) disynaptic basin that characterise the C4–C5 double bond of DCE **6** remain at the DCE moiety of **MC2-o** with a total population of 3.26 e. At **MC2-o**, there is already a notable GEDT, 0.14 e.

At **TS2-o**, d(C1–C4) = 2.311 Å and d(N3–C5) = 2.093 Å, the two V(C4,C5) and V’(C4,C5) disynaptic basins describing the C4–C5 bonding region of the DCE framework have merged into one single V(C4,C5) disynaptic basin as a consequence of the slight depopulation of this region by 0.07 e. Coming from **MC2-o** to **TS2-o**, the populations of the V(C1,N2) and V(N2,N3) disynaptic basins have remained almost invariable, while those corresponding to the V(C1) and V(N3) monosynaptic basins have increased and decreased to 0.53 e and 3.17 e, respectively. At **TS2-o**, the GEDT has increased to 0.25 e, a value that allows establishing the polar character of the *ortho* regioisomeric channel of this 32CA reaction.

The changes in electron density taking place coming from **MC2-o** to **TS2-o**, which are associated with slight electron density variations within the molecular system mainly directed towards the rupture of the C4–C5 double bond of DCE **6**, population of the C1 *pseudoradical* center and decrease of the non-bonding N3 electron density,

account for the very low activation barrier of *ca.* 0.6 kcal·mol$^{-1}$ from **MC2-o** (see Table S5).

At **P1-o**, d(C1–C4) = 2.121 Å and d(N3–C5) = 2.560 Å, a new V(C4) monosynaptic basin, integrating 0.07 e, is found at the C4 carbon of the DCE framework while a slight depopulation of the V(C4,C5) disynaptic basin to 3.15 e can be noticed (see Table S5 and **P1-o** in Figure S5). This new V(C4) monosynaptic basin is related with the C4 *pseudoradical* center demanded for the subsequent C1–C4 bond formation. At this point, the V(C1) monosynaptic basin has reached 0.69 e and the V(N3) one has continued being depopulated to 3.05 e. At **P1-o**, the GEDT is very high 0.32 e.

At **P2-o**, d(C1–C4) = 2.048 Å and d(N3–C5) = 2.536 Å, the disappearance of the two V(C1) and V(C4) monosynaptic basins present at the previous **P1-o**, together with the presence of a new V(C1,C4) disynaptic basin integrating 0.96 e, indicates that the formation of the first C1–C4 single bond starts at a C–C distance of 2.05 Å through the C-to-C coupling of two C1 and C4 *pseudoradical* centers (see **P2-o** in Figure S5) [1]. Another relevant topological change than can be noticed at this point is the existence of a new V(N2) monosynaptic basin, integrating 0.61 e, associated with the non-bonding N2 nitrogen lone pair of the final pyrazolidinone **7**, as a consequence of the strong depopulation of the adjacent V(C1,N2) disynaptic basin to 2.40 e. At **P2-o**, coinciding with the formation of the first C1–C4 single bond, the GEDT has reached the maximum value among the selected points of the IRC, 0.35 e. Yet again, this high value emphasises the high polar character of the 32CA reaction between AI **1b** and DCE **6**.

At **P3-o**, d(C1–C4) = 1.556 Å and d(N3–C5) = 1.865 Å, a new V(C5) monosynaptic basin, associated with a C5 *pseudoradical* center, can be observed at the DCE framework integrating 0.63 e (see **P3-o** in Figure S5). This electron density mainly comes from the strong depopulation that the V(C4,C5) disynaptic basin has suffered to 2.62 e. At the same time, the V(N3) monosynaptic basin present at the previous **P3-o** splits into two new V(N3) and V'(N3) monosynaptic basins integrating 2.62 e and 0.32 e (Figure S5). While the first one is associated to the N3 nitrogen lone pair, the second one can be related to the N3 *pseudoradical* center demanded for the subsequent N3–C5 single bond formation. On the other hand, the V(C1,C4) disynaptic basin has acquired 1.81 e, and the V(N2) monosynaptic basin increases its population to 2.00 e as a consequence of the depopulation of the neighbouring V(C1,N2) and V(N2,N3)

disynaptic basins to 1.78 e and 1.58 e. At **P3-o**, the GEDT has decreased to 0.14 e due to a retro-donation process from the DCE to the AI substructures.

At **P4-o**, d(C1−C4) = 1.554 Å and d(N3−C5) = 1.837 Å, while the two V(N3) and V(C5) monosynaptic basin have disappeared, a new V(N3,C5) disynaptic basin is created with an electron population of 1.06 e (see **P4-o** in Figure S5). This topological feature indicates that the formation of the second N3−C5 single bond begins at an N−C distance of *ca.* 1.84 Å through the C-to-N coupling of two C5 and N3 *pseudoradical* centers. At this point, the population of the first formed V(C1,C4) disynaptic basin remains *ca.* 96% of that at pyrazolidinone **7**, thus characterising a *two-stage one-step* mechanism [2]. At **P4-o**, the GEDT is 0.13 e.

Finally, at pyrazolidinone **7**, d(C1−C4) = 1.515 Å and d(N3−C5) = 1.474 Å, only electron density variations are noticeable until the molecular system is completely relaxed (see electron populations in Table S5). Similar to **8**, the new V(C1,C4) and V(N3,C5) disynaptic basins have reached populations of 1.89 e and 1.70 e and, interestingly, the V(N2,N3) disynaptic basin ends with a very low population of 1.36 e. At **7**, the GEDT is almost null, −0.01 e.

**Table S5**. Valence basin populations N calculated from the ELF of the stationary points and some points of the IRC involved in the formation of the new C1–C4 and N3–C5 single bonds along the less favourable *ortho* regioisomeric channel associated with the polar 32CA reaction between AI **1b** and DCE **6**. Distances are given in Å, GEDT values and electron populations in e, and relative[a] energies in kcal·mol$^{-1}$.

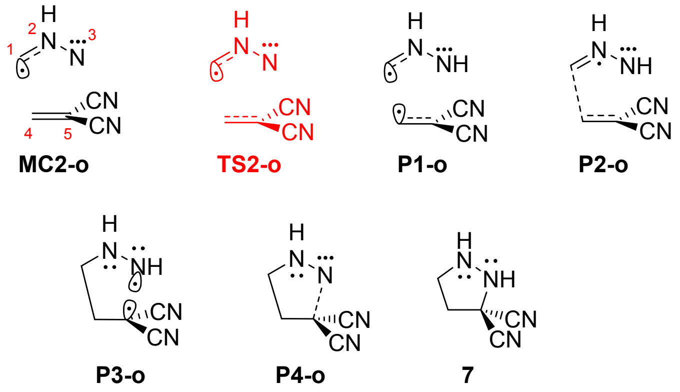


| Points | **1b** | **6** | **MC2-o** | **TS2-o** | **P1-o** | **P2-o** | **P3-o** | **P4-o** | **7** |
|---|---|---|---|---|---|---|---|---|---|
| d(C1–C4) | | | 2.758 | 2.311 | 2.121 | 2.048 | 1.556 | 1.554 | 1.515 |
| d(N3–C5) | | | 2.752 | 2.625 | 2.560 | 2.536 | 1.865 | 1.837 | 1.474 |
| ΔE | | | -6.3 | -5.7 | -6.5 | -7.5 | -37.9 | -39.2 | -56.2 |
| GEDT | | | 0.14 | 0.25 | 0.32 | 0.35 | 0.14 | 0.13 | -0.01 |
| V(C1,N2) | 2.95 | | 2.98 | 2.97 | 2.96 | 2.40 | 1.78 | 1.77 | 1.75 |
| V(N2) | | | | | | 0.61 | 2.00 | 2.02 | 2.26 |
| V(N2,N3) | 2.10 | | 2.12 | 2.15 | 2.12 | 2.10 | 1.58 | 1.58 | 1.36 |
| V(C4,C5) | | 1.66 | 1.64 | 3.19 | 3.15 | 3.01 | 1.99 | 1.99 | 1.82 |
| V'(C4,C5) | | 1.66 | 1.62 | | | | | | |
| V(C1) | 0.31 | | 0.38 | 0.53 | 0.69 | | | | |
| V'(C1) | 0.31 | | | | | | | | |
| V(N3) | 3.53 | | 3.36 | 3.17 | 3.05 | 2.92 | 2.62 | 2.60 | 2.25 |
| V'(N3) | | | | | | | 0.32 | | |
| V(C4) | | | | | 0.07 | | | | |
| V(C5) | | | | | | | 0.72 | | |
| V(C1,C4) | | | | | | 0.96 | 1.81 | 1.81 | 1.89 |
| V(N3,C5) | | | | | | | | 1.06 | 1.70 |

[a] Relative to the separated reagents AI **1b** and DCE **6**.

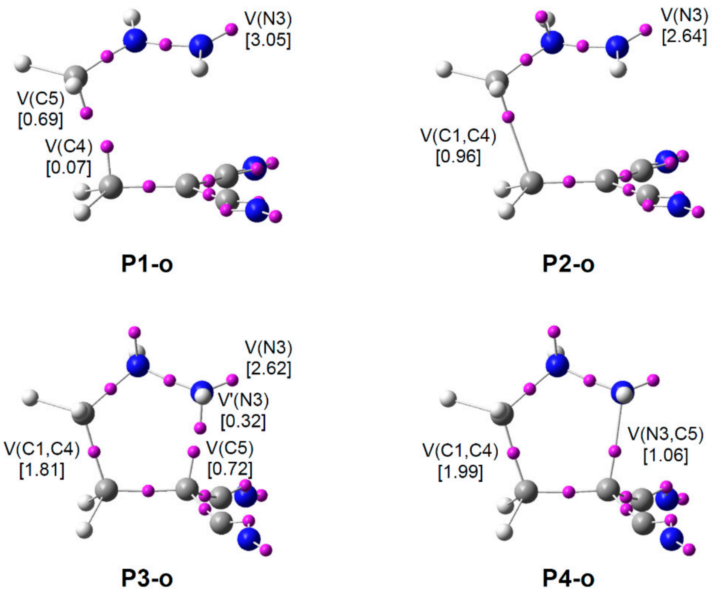


**Figure S5**. ELF attractor positions for the selected points of the IRC involved in the formation of the C1–C4 and N3–C5 single bonds along the less favourable *ortho* reactive channel associated with the polar 32CA reaction between AI **1b** and DCE **6**. The electron populations, in e, are given in brackets.

## 3. Theoretical background

### *3.1 Topological analysis of the ELF*

Like many other chemical concepts, chemical bonds are defined in a rather ambiguous manner as they are not observable, but rather belong to a representation of the matter at a microscopic level, which is not fully consistent with quantum mechanical principles. To harmonise the chemical description of matter with quantum chemical postulates, several mathematical models have been developed. Among them, the theory of dynamical systems [3], convincingly introduced by Bader through the Theory of Atoms in Molecules (AIM) [4], has become a powerful method of analysis. The AIM theory enables a partition of the electron density within the molecular space into basins associated with atoms. Another appealing procedure that provides a more straightforward connection between the electron density distribution and the chemical structure is the quantum chemical analysis of the ELF of Becke and Edgecombe [5]. ELF constitutes a useful relative measure of the electron pair localisation characterising the corresponding electron density [6,7]. Within the framework of Density Functional Theory (DFT), ELF is a density-based property that can be interpreted in terms of the positive-definite local Pauli and Thomas Fermi kinetic energy densities in a given system. In the validity of such a framework, these quantities provide key information to evaluate the relative local excess of kinetic energy density associated to the Pauli principle. ELF presents values in the range [3]; the highest values being associated with the spatial positions with higher relative electron localisation [8-11]. After an analysis of the electron density, ELF provides basins of attractors, which are the domains in which the probability of finding an electron pair is maximal. The spatial points in which the gradient of ELF has a maximum value are designated as attractors [10]. ELF basins are classified as core basins, C(...), and valence basins, V(...). The latter are characterised by the synaptic order, *i.e.* the number of atomic valence shells in which they participate. Thus, there are monosynaptic, disynaptic, trisynaptic basins and so on [11]. Monosynaptic basins, labelled V(A), correspond to the lone pairs or non-bonding regions, while disynaptic basins, labelled V(A,B), connect the core of two nuclei A and B and, thus, correspond to a bonding region between A and B. This description recovers the Lewis bonding model, providing a very suggestive graphical representation of the molecular system.

*3.2 BET*

When trying to achieve a better understanding of bonding changes in organic chemical reactions, the so-called BET has proved to be a very useful methodological tool [12]. BET applies Thom's Catastrophe Theory (CT) concepts [13-15] to the topological analysis of the gradient field of the ELF.

Within the BET methodology [12], the structural stability of the critical points of the ELF gradient field is examined for the system of nuclei and electrons 'evolving' along the Born-Oppenheimer energy hypersurface or a given reduced reaction coordinate, *e.g.* the intrinsic reaction coordinate, occurring as a result of the variation in the control space parameters from reactive to product configurations. The chemical process becomes thus rationalised in terms of successive structural stability domains (SSDs), also called phases, comprising structures along the path where the number and type, *e.g.* synaptic orders, of critical points of the gradient field of ELF remain without changes [12].

Within the BET context, the turning points between these phases are located and the discontinuities or bifurcation catastrophes can be identified. BET allows, thus, characterising unequivocally the behaviour of the dynamical system upon bifurcations associated with the ELF gradient field changing along the reaction coordinate. The different catastrophes in this case correspond to the reduction or the increase of the critical points associated with attractors of electron pairs defining bonding and non-bonding, *e.g.* lone pairs, domains for electron (de)localisation.

A detailed examination of the topology of ELF along the IRC pathway for a given reaction reveals the existence of several catastrophes belonging exclusively to the fold, $F$ and $F^{\dagger}$, and cusp, $C$ and $C^{\dagger}$, elementary types, according to Thom's classification. The $F$ catastrophe merges an attractor and a saddle point into a wandering point, *i.e.* a non-critical point, decreasing the number of basins by 1, whereas $F^{\dagger}$ splits a wandering point into an attractor and a saddle point increasing the number of basins by 1. The $\dagger$ superscript is used in those catastrophes in which either the number of attractors or the synaptic order increase. The cusp catastrophe $C$ merges two attractors and a saddle point into an attractor decreasing the number of basins by 1, while $C^{\dagger}$ splits an attractor into two attractors and a saddle point increasing the number of basins by 1. The symbol of a catastrophe written in bold is used to mark a catastrophe leading to the formation of the first covalent bond. The analysis of the changes in the number and type of ELF valence

basins for the structures involved along the IRC of the reaction allows establishing a set of points, **Pi**, separating the different phases that characterise the studied molecular mechanism.

Several theoretical studies have shown that the topological analysis of the ELF offers a suitable framework for the study of the changes of electron density [16-22]. This methodological approach is used as a valuable tool to understand the bonding changes along the reaction path and, consequently, to establish the nature of the electronic rearrangement associated with a given molecular mechanism within the BET perspective.

## 4. References


1 Domingo, L. R. A New C-C Bond Formation Model Based on the Quantum Chemical Topology of Electron Density. *RSC Adv.* **2014**, *4*, 32415–32428.

2 Domingo, L. R.; Saéz, J. A.; Zaragozá, R. J.; Arnó. M. Understanding the Participation of Quadricyclane as Nucleophile in Polar [2σ+2σ+2π] Cycloadditions toward Electrophilic π Molecules. *J. Org. Chem.* **2008**, *73*, 8791-8799.

3 Abraham, R. H.; Shaw, C. D. *Dynamics: The Geometry of Behavior*, Addison-Wesley, Redwood City, CA, 1992.

4 Bader, R. F. W. *Atoms in Molecules. A Quantum Theory*, Claredon Press, Oxford, U.K, 1990.

5 Beck, A. D.; Edgecombe. K. E. A simple measure of electron localization in atomic and molecular systems. *J. Chem. Phys.* **1990**, *92*, 5397–5403.

6 Silvi, B.; Savin, A. Classification of chemical bonds based on topological analysis of electron localization functions. *Nature* **1994**, *371*, 683−686.

7 Savin, A.; Silvi, B.; Colonna. F. Topological analysis of the electron localization function applied to delocalized bonds. *Can. J. Chem.* **1996**, *74*, 1088–1096.

8 Savin, A.; Becke, A. D.; Flad, J.; Nesper, R.; Preuss, H.; Vonschnering. H. G. A new look at electron localization. *Angew. Chem. Int. Ed.* **1991**, *30*, 409–412.

9 Savin, A.; Nesper, R.; Wengert, S.; Fassler, T. F. ELF: The Electron Localization Function. *Angew. Chem. Int. Ed.* **1997**, *36*, 1809–1832.

10 Savin. A. On the significance of ELF basins. *J. Chem. Sci.* **2005**, *117*, 473–475.

11 Silvi. B. The synaptic order: A key concept to understand multicenter bonding. *J. Mol. Struct.* **2002**, *614*, 3–10.

12 Krokidis, X.; Noury, S.; Silvi, B. Characterization of elementary chemical processes by catastrophe theory. *J. Phys. Chem. A* **1997**, *101*, 7277–7282.

13 Thom, R. *Structural Stability and Morphogenesis: An Outline of a General Theory of Models*, Inc., Reading, Mass (London-Amsterdam, 1976).

14 Woodcock, A.E.R.; Poston, T. *A Geometrical Study of Elementary Catastrophes,* (Spinger-Verlag, Berlin, 1974).

15 Gilmore, R. *Catastrophe Theory for Scientists and Engineers* (Dover, New York, 1981).

16 Berski, S.; Andrés, J.; Silvi, B.; Domingo, L.R. The joint use of catastrophe theory and electron localization function to characterize molecular mechanisms. A density

functional study of the Diels-Alder reaction between ethylene and 1,3-butadiene. *J. Phys. Chem. A* **2003**, *107*, 6014–6024.

17 Chamorro. E. The nature of bonding in pericyclic and pseudopericyclic transition states: Thermal chelotropic decarbonylations. *J. Chem. Phys.* **2003**, *118*, 8687–8698.

18 Polo, V.; Andrés, J.; Berski, S.; Domingo, L.R.; Silvi, B. Understanding reaction mechanisms in organic chemistry from catastrophe theory applied to the electron localization function topology. J. Phys. Chem. A **2008**, *112*, 7128–7136.

19 Domingo, L. R.; Chamorro, E.; Pérez P. Understanding the Reactivity of Captodative Ethylenes in Polar Cycloaddition Reactions. A Theoretical Study. *J. Org. Chem.* **2008**, *73*, 4615–4624.

20 Domingo, L. R.; Chamorro, E.; Pérez. P. Understanding the mechanism of non-polar Diels–Alder reactions. A comparative ELF analysis of concerted and stepwise diradical mechanisms. *Org. Biomol. Chem.* **2010**, *8*, 5495–5504.

21 Berski, S.; Ciunik, Z. The mechanism of the formation of the hemiaminal and Schiff base from the benzaldehyde and triazole studied by means of the topological analysis of electron localisation function and catastrophe theory. *Mol. Phys.* **2015**, *113*, 765–781.

22 Ríos-Gutiérrez, M.; Pérez, P.; Domingo. L. R. A bonding evolution theory study of the mechanism of [3+2] cycloaddition reactions of nitrones with electron-deficient ethylenes. *RSC Adv.* 2015, **5**, 58464–58477.
